# DNMT Inhibitors Increase Methylation in the Cancer Genome

**DOI:** 10.3389/fphar.2019.00385

**Published:** 2019-04-24

**Authors:** Anil K. Giri, Tero Aittokallio

**Affiliations:** ^1^Institute for Molecular Medicine Finland, University of Helsinki, Helsinki, Finland; ^2^Helsinki Institute for Information Technology, Department of Computer Science, Aalto University, Espoo, Finland; ^3^Department of Mathematics and Statistics, University of Turku, Turku, Finland

**Keywords:** DNA methyltransferase inhibitors, decitabine, azacytidine, anticancer treatment, olfactory receptor pathway, alternative splicing

## Abstract

DNA methyltransferase inhibitors (DNMTi) decitabine and azacytidine are approved therapies for myelodysplastic syndrome and acute myeloid leukemia, and their combinations with other anticancer agents are being tested as therapeutic options for multiple solid cancers such as colon, ovarian, and lung cancer. However, the current therapeutic challenges of DNMTis include development of resistance, severe side effects and no or partial treatment responses, as observed in more than half of the patients. Therefore, there is a critical need to better understand the mechanisms of action of these drugs. In order to discover molecular targets of DNMTi therapy, we identified 638 novel CpGs with an increased methylation in response to decitabine treatment in HCT116 cell lines and validated the findings in multiple cancer types (e.g., bladder, ovarian, breast, and lymphoma) cell lines, bone marrow mononuclear cells from primary leukemia patients, as well as peripheral blood mononuclear cells and ascites from platinum resistance epithelial ovarian cancer patients. Azacytidine treatment also increased methylation of these CpGs in colon, ovarian, breast, and lymphoma cancer cell lines. Methylation at 166 identified CpGs strongly correlated (|r|≥ 0.80) with corresponding gene expression in HCT116 cell line. Differences in methylation at some of the identified CpGs and expression changes of the corresponding genes was observed in TCGA colon cancer tissue as compared to adjacent healthy tissue. Our analysis revealed that hypermethylated CpGs are involved in cancer cell proliferation and apoptosis by P53 and olfactory receptor pathways, hence influencing DNMTi responses. In conclusion, we showed hypermethylation of CpGs as a novel mechanism of action for DNMTi agents and identified 638 hypermethylated molecular targets (CpGs) common to decitabine and azacytidine therapy. These novel results suggest that hypermethylation of CpGs should be considered when predicting the DNMTi responses and side effects in cancer patients.

## Introduction

DNA methyltransferase inhibitors (DNMTi) are widely used as chemical tools for hypomethylating the genome, with an aim to understand the role of DNA methylation in multiple processes (e.g., X-chromosome inactivation and DNA imprinting) and as an anti-cancer therapy (Minkovsky et al., 2015; Ramos M.P. et al., 2015; Bohl et al., 2018). At present, two DNMTi drugs, decitabine and azacytidine, have been approved for treating patients with myelodysplastic syndrome (MDS) and acute myeloid leukemia (AML) (Döhner et al., 2017; Bohl et al., 2018), and are also being tested as therapeutic options in multiple solid cancers (Fu et al., 2011; Singal et al., 2015; Lee et al., 2018). However, treatment response rates have remained very low in both solid and hematological cancers patients (Derissen et al., 2013; Nervi et al., 2015; Koch et al., 2018). At the same time, DNMTi treatment incurs considerable side effects (e.g., bleeding, anemia, and joint pain). Attempts are being made to improve treatment efficacy (e.g., by testing combinations of DNMTis with other anticancer agents) and safety (e.g., by stratifying cancer patients into DNMTi responders and non-responders) (Nervi et al., 2015) of the drugs. However, the precise prediction of drugs that show synergy with DNMTi in combination as well as distinguishing the drug responders from the non-responders are challenging tasks and are currently compromised by our incomplete knowledge of the drug mechanisms of action in various cancer-types (Ramos F. et al., 2015; Wang et al., 2018).

Hypomethylation of the genome is one of the principal mechanisms behind the therapeutic benefit of DNMTi treatment and hypomethylated targets of the drugs (e.g., CpGs, genes, genomic region, pathways) have been functionally well-characterized in multiple cancer cell lines and other model systems (Sarkar et al., 2013; Tobiasson et al., 2017; Koch et al., 2018). However, DNMTi induced hypomethylation of the genome is not sufficient to clearly understand DNMTis’ response and side effects (Ramos F. et al., 2015; Wang et al., 2018), suggesting presence of additional mechanisms by which DNMTis’ may modulate their responses. Therefore, there is a critical need to better understand the mechanism of action of DNMTis’ in more details, including their molecular targets in various cancer cells. Only a few studies indicated that DNMTi treatment can also lead to DNA hypermethylations in specific cell types, probably due to an increased expression of DNA methylating enzymes (Broday et al., 1999; Kastl et al., 2010; Chowdhury et al., 2015). For example, Kastl et al. (2010) reported an increase in the mRNA level of DNMT1, DNMT3a, and DNMT3b genes in docetaxel-resistant MCF7 cells, as compared to drug-sensitive cells when treated with decitabine. However, we currently lack the information of the genomic location and function of molecular targets in the cancer genome that can resist the DNA demethylation and show hypermethylation in response to DNMTi treatment.

In the present work, we hypothesized that DNMTi treatment causes hypermethylation in the genome, and therefore we systematically investigated the extent of hypermethylated CpGs, their location, and biological role by analyzing cell line and primary tumor data. Since colon cancer tissue has revealed silencing of various tumor suppressor genes due to hypermethylation (Huidobro et al., 2012), and especially the HCT116 cell line has widely been utilized to study DNA methylation and its role in regulating gene expression in colon cancer (De Carvalho et al., 2012; Yang et al., 2014), we selected HCT116 cell line as our primary disease model to discover novel hypermethylated CpGs. We validated the findings in a panel of 53 other cancer cell lines (e.g., lymphoma, colon, ovarian, and breast cancer), as well as in bone marrow cells of 2 primary leukemia patients, 2 PBMCs and 2 ascites samples of ovarian cancer patients. There was a significant correlation in the methylation of a fraction of identified CpGs and corresponding genes’ expression in HCT116 cell line indicating that the identified hypermethylated CpGs are clinically functional. Our work aims at initiating the search of hypermethylation in the response to DNMTi treatment in cancer cells and tissues as a novel mechanism by which one could predict in future the therapeutic and adverse effects of DNMTis in multiple cancer sub-types (i.e., stratified medicine), or even in individual cancer patients (i.e., personalized medicine).

## Materials and Methods

### Processing of Published Methylation Data

To identify CpGs with increased methylation after decitabine treatment, we re-analyzed the DNA methylation (Illumina 450K platform, GSE51810) data from the study of Yang et al. (2014), where colon cancer HCT116 cells were treated with decitabine (0.3 mM) for 72 h. Cells were maintained in McCoy’s 5A medium, supplemented with 10% fetal bovine serum along with 1% penicillin/streptomycin after drug treatment, and followed through 5, 14, 24, 42, and 68 days. The increase in DNA methylation in HCT116 cells were validated using methylation data from the study of Han et al. (2013) (Illumina 450K, GSE41525), where HCT116 and T24 bladder cancer cell lines were treated with 0.3 and 1 μM of decitabine, respectively, for 24 h, and Illumina 450K assay was performed for both untreated and decitabine-treated cells. We also tested the increase in DNA methylation of identified CpGs using DMSO (as mock treatment) and decitabine-treated (0.06 μM for 72 h) MCF7 breast cancer cells using data generated by Leadem et al. (2018) (Illumina 450K platform, GSE97483). These cells were cultured in Minimum Essential Medium (MEM) with 10% fetal bovine serum).

Further, we validated our findings identified in cell lines using DNA methylation data from primary bone marrow mononuclear cells (Illumina 450K platform, GSE20945-GPL13534) from two freshly diagnosed acute myelogenous leukemia patients (IDs 1107 and 1307) and one healthy control (BM) (Tsai et al., 2012). The frozen primary cells were thawed and cultured in Poietics HPGM supplemented with 50 ng/ML human thrombopoietin, 25 ng/mL stem cell factor, 50 ng/mL Flt-3 ligand, 10 ng/mL recombinant human IL-3, 10 ng/mL recombinant human IL-6, 10 ng/mL recombinant human GM-CSF and 1 ng/mL recombinant human G-CSF, as specified in Tsai et al. (2012). These cells have been treated with 10 nM of decitabine or PBS (control) for 72 h with daily medium change. DNA methylation for patient 1307 was assessed at day 3 as well as day 14 and for patient 1107 at day 7. We also analyzed genome wide DNA methylation (Illumina Infinium 27K Beadchip, GSE31826) profile of 14 peripheral blood mononuclear cells (PBMC), 8 tumor biopsies and 6 ascites samples from recurrent, platinum resistant epithelial ovarian cancer patients, before and after treatment with decitabine (Matei et al., 2012). Patients have been treated with decitabine (Eisai) at 10 mg/m^2^ given intravenously daily for 5 days. The methylation for each sample was measured before treatment at day 1 (control) and after decitabine treatment at day 8 (treated). The comparison of treated and control samples was done using the paired Wilcoxon non-parametric test on the basis of 16 common CpGs (out of 638) with hypermethylation.

We extended our discovery analyses also to another DNMTi inhibitor, azacytidine, by re-analyzing DNA methylation data (Illumina 450K, GSE45707) for untreated and azacytidine-treated (5 mM for 72 h) lymphoma U937 cell line. We further re-analyzed additional methylation (Illumina 450K platform, GSE57342) and gene expression data (GSE57343) from 26 breast cancer cell lines (MDA231, SKBR3, HCC38, ZR7530, HCC1937, CAMA1, MDA415, HCC1500, BT474, EFM192A, MDA175, MDA468, MDA361, HCC1954, BT20, ZR751, HCC1569, EFM19, T47D, MDA453, MCF7, HCC1187, HCC1419, MDA436, SUM149, and SUM159), 12 colorectal cancer cell lines (SW48, HCT116, HT29, RKO, SW480, Colo320, Colo205, SW620, SNUC-1, CACO-2, SK-CO1, and Colo201), and 13 ovarian cancer cell lines (TykNu, CAOV3, OAW28, OV2008, ES2, EF27, Kuramochi, OVKATE, Hey, A2780, OVCAR3, OVCAR5, and SKOV3) measured after mock treatment and 0.5 μM azacytidine treatment for 72 h (Li et al., 2014). The cells were cultured and maintained under recommended conditions for each cell line (Li et al., 2014).

To investigate the alteration in methylation status of identified probes in cancerous tissue and their role in gene expression regulation, TCGA level 3 HumanMethylation 450K data and normalized RNA-seq gene expression profiles for colon adenocarcinoma (COAD) patients (both tumor and adjacent healthy tissue samples) were downloaded using the FireBrowse tool^1^.

In all the analysis, methylation status at a CpG site was measured as beta value (β), which is the ratio of the methylated probe intensity and the overall intensity (sum of methylated and unmethylated probe intensities designed for a particular CpG in 450K beadchip). β ranges from 0 to 1, indicating no methylation (β = 0) to complete methylation of the CpGs (β = 1). We performed appropriate quality control of the published data before their downstream analysis as described previously (Giri et al., 2017). As a part of quality control, we removed all the CpGs with missing values and CpGs assessed by probes that have a tendency of cross-hybridization, as specified in the supplementary file of Chen et al. (2013). To remove any possible bias due to design differences in the type of probes (the type I and type II probes) present in the Illumina 450K platform, we performed BMIQ normalization (Teschendorff et al., 2013) to the DNA methylation data from the TCGA samples before correlation and differential methylation analysis. All other data processing was done using local inbuilt commands in R.

### Identification of Probes With Increased Methylation in HCT116 Cell Line

We calculated the difference in methylation level of CpGs before and after treatment with decitabine in HCT116 cell line at day 5 in data from Yang et al. (2014) (GSE51810). CpG that showed an increase in β-value of greater than or equal to 0.10 (Δβ ≥ 0.10) between untreated control and decitabine treated HCT116 cells were identified as hypermethylated CpGs. The population doubling time reported by Yang et al. (2014) was used for correlation analysis with methylation level of identified CpGs. We estimated population doubling time as 24 h for untreated cells and 34, 38, 31, 28, 26, and 25.5 h for treated cells at time points of 5, 14, 24, 42, and 68 days.

### Gene Expression Data Analysis

We downloaded gene expression profiles of untreated control and decitabine treated HCT116 cell lines at 5, 14, 24, and 42 days (GSE51811, Illumina HumanHT-12 V4.0 expression beadchip) from the same study of Yang et al. (2014). The downloaded data were log_2_-transformed and normalized using robust spline normalization (RSN) method as implemented in the lumi package in R (Du et al., 2008). Pearson correlation between methylation and gene expression across different time points in HCT116 cell line was assessed using the cor.test function of R.

Normalized RNA-seq data of TCGA colon adenocarcinoma patients downloaded from FireBrowse tool^1^ were further normalized using voom function in the limma package (Ritchie et al., 2015), and these data were Z-transformed before the differential and correlation analyses. Wilcoxon non-parametric test was used to identify differentially methylated and differentially expressed genes between TCGA adenocarcinoma tumors and adjacent healthy tissue samples (FDR < 0.05). Only those adenocarcinoma samples that had both DNA methylation and gene expression information were used for the correlation analyses.

In order to find the possible reasons for absence of hypermethylation by low dose treatment with azacytidine in breast cancer cell lines, we extracted and compared the gene expression DNMT3A, DNMT3B, DNMT1, and DNMTL across 26 breast cancer, 12 colorectal cancer, and 13 ovarian cancer cell lines at day 3 (after azacytidine treatment) using gene expression data from Li et al. (2014) (GSE57343). We downloaded the processed gene expression data of azacytidine treated cells normalized to mock treated respective controls and compared across cell lines of different tissue types.

### Gene Annotation and Pathway Enrichment Analysis

Identified CpGs were annotated for their location in the genome based on annotation file provided by Illumina^2^. Gene ontology and pathway enrichment analysis of the genes corresponding to CpGs with hypermethylation were done using GeneCodis (Tabas-Madrid et al., 2012). Statistical enrichment was assessed by FDR corrected *p*-values from hypergeometric test for separate ontology terms and pathways. GENEMANIA (Zuberi et al., 2013) was used to construct and visualize the interaction network between genes and the transcription factor regulating them. Key term enrichment analysis for the genes corresponding to CpGs was done using DAVID (Huang Da et al., 2009).

## Results

### DNMTi Treatment Increases Methylation of a Small Portion of the CpGs

**FIGURE 1:**
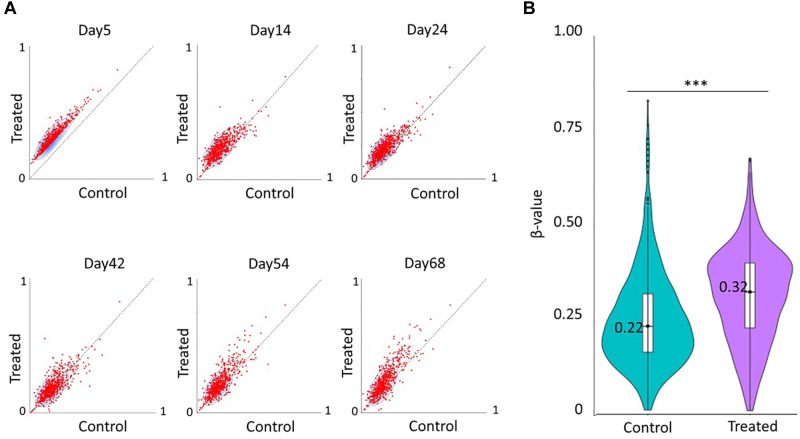
Decitabine treatment increases DNA methylation levels of a subset of CpGs. **(A)** Scatter plots showing DNA methylation patterns of 638 differentially methylated CpGs between untreated control cells (x-axis) and decitabine-treated cells (y-axis) at various time points in the study of Yang et al. (2014). **(B)** Violin plot showing the median methylation level (horizontal line) and distribution patterns (density and IQR) of the identified 583 CpGs in untreated and 0.3 μM decitabine-treated HCT116 cells after 24 h in the study of Han et al. (2013). The statistical significance was assessed using the non-parametric Wilcoxon test. ^∗∗∗^*p* < 0.0005.

After quality control, we re-analyzed a total of 369886 CpGs across the genome of HCT116 colon cancer cells from Yang et al. (2014), and identified hypermethylation (Δβ ≥ 0.10) of 638 CpGs in 393 unique genes after 5 days of decitabine treatment (0.3 mM for 72 h), as compared to untreated cells (Figure 1A). Most of the loci were hypomethylated in the genome (median β = 0.18), and after decitabine treatment, a median increase of 0.12 (Δβ = 0.12, *p* < 0.0005) in methylation level was observed for these sites. The detailed list of the identified CpGs is provided in Supplementary Table 1. Change in methylation at 34 of the identified CpGs were strongly correlated (Pearson correlation coefficient > 0.80, FDR <0.05) with the population doubling time of HCT116 cell lines after decitabine treatment (Supplementary Table 2), indicating that methylation at a fraction of identified CpGs affects proliferation and growth of cancer cells. However, most of the identified sites loss their hypermethylation by day 10 (Figure 1) suggesting that the observed hypermethylation is transient. Re-analysis of another methylation data for HCT116 cell line from the Han et al. (2013) study validated our finding, as we found a corresponding increase in methylation level (median Δβ = 0.09) at 583 common CpGs after decitabine treatment (0.3 μM for 24 h) (Figure 1B). These results indicate that the increase in DNA methylation at most of the identified sites starts as early as 24 h after the DNMTi treatment and lasts up to at least day 5. The result suggests that there are CpGs that not only resist the demethylation in response to DNMTi but also show transient hypermethylation.

**FIGURE 2:**
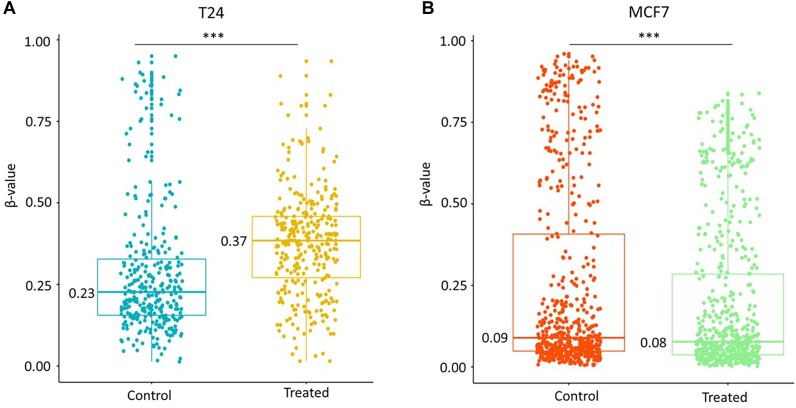
Increase in methylation of identified CpGs is cell line-specific. **(A)** Methylation level of 616 identified probes common in untreated and decitabine-treated (1 μM for 24 h) bladder cancer T24 cell line. An increase in median DNA methylation levels (median Δβ = 0.14) at 616 common CpGs was observed after the drug treatment. **(B)** Methylation level of 590 identified probes common in mock (DMSO) and decitabine-treated (0.06 μM for 72 h) breast cancer MCF7 cell line. We observed a smaller decrease in methylation level (median Δβ = –0.01, *p* < 0.005) when the cells were treated with lower dose (0.06 μM) of decitabine in MCF7 cell lines. The statistical significance was assessed using the non-parametric Wilcoxon test. ^∗∗∗^*p* < 0.0005.

Further, we also tested the effect of decitabine on identified differentially methylated CpGs in a bladder cancer cell line (T24). An increase in median DNA methylation levels (median Δβ = 0.14, *p* < 0.0005) at 616 common CpGs was observed after the drug treatment (1 μM for 24 h) in T24 cells (Figure 2A) in contrast to a significant decrease in the methylation level of other CpGs present in the 450K beadchip (median Δβ = −0.14) as shown in Supplementary Figure 1. However, we did not observe any increase in methylation level of 590 common identified CpGs (median Δβ = −0.01, *p* < 0.0005) in breast cancer MCF7 cell line treated with 0.06 μM of decitabine for 72 h (Figure 2B). Replication of the results in multiple cancer cell lines indicates that hypermethylation in the cancer genome is a common effect of decitabine treatment that can contribute to DNMTis’ response.

**FIGURE 3:**
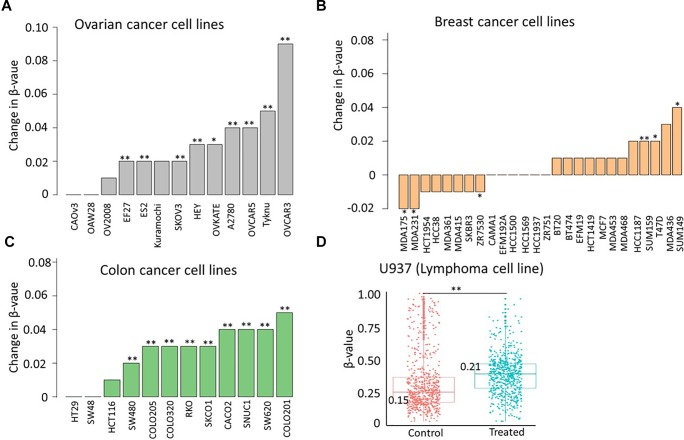
Azacytidine treatment increases methylation of identified sites in a subset of cell lines. Change in median methylation level of identified CpGs in **(A)** 13 ovarian cancer cell lines **(B)** 26 breast cancer cell lines, and **(C)** 12 colon cancer cell lines. The bar plot represents the difference in the median methylation level of identified CpGs between cells treated with 0. 5 μM azacytidine (test group) or carboplatin (mock group) after 72 h. **(D)** Scatter plot showing the distribution of methylation level of identified probes in untreated (control) and treated cells (5 mM of azacytidine for 72 h) in U937 lymphoma cell lines. The statistical significance was assessed using the non-parametric Wilcoxon test. ^∗^*p* < 0.05, ^∗∗^*p* < 0.005.

To study the effect of another DNMT inhibitor, azacytidine, on methylation of identified CpGs, we next re-analyzed 450K data from 52 pan-cancer cell lines. We found a significant increase in methylation of identified CpGs in 9 out of 13 ovarian cancer cell lines (median Δβ > 0.03), 3 out of 26 breast cancer cell lines (median Δβ > 0.01), and in 9 out of 12 colon cancer cell lines (median Δβ > 0.02), and in 1 lymphoma cell line (U937, median Δβ > 0.06) after azacytidine treatment (Figure 3A–D). Validation of the observed hypermethylation at identified target CpGs in multiple cancer types (bladder, ovarian, breast, and lymphoma cancer cell lines) suggests that hypermethylation in the genome is a shared phenomenon in response to multiple DNMTi agents and that the degree of the hypermethylation is cancer- and cell line-specific.

### Identified CpGs Show Trend of Hypermethylation in AML and Ovarian Cancer Primary Cells

**FIGURE 4:**
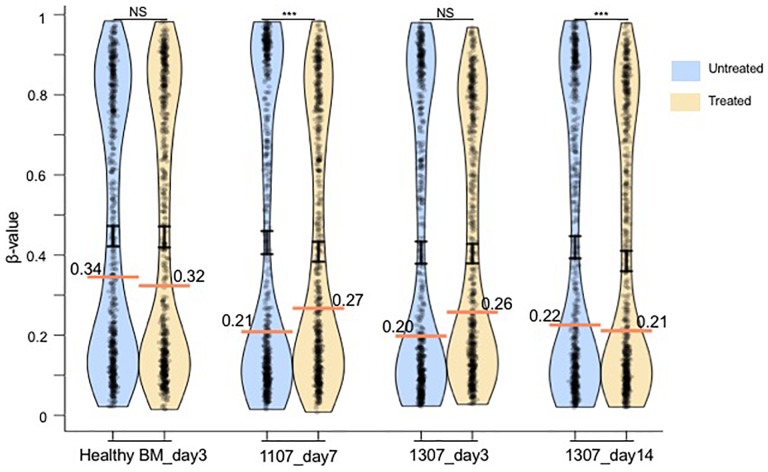
Pirate plot show the median methylation level (horizontal colored line) and distribution pattern (mean ± standard error as vertical black line) of the identified 638 CpGs in untreated and treated (10 nM of decitabine) mononuclear cells from a healthy bone marrow (BM) sample and two primary leukemia patient samples (IDs 1307 and 1107), based on the data from the study of Tsai et al. (2012). DNA methylation assay for the patient 1307 was performed at days 3 and 14. For the patient 1107, the assay was performed only at day 7. The median methylation level is indicated in each plot. The statistical significance was assessed using the paired non-parametric Wilcoxon test. NS, not significant. ^∗∗∗^*p* < 0.0005.

To investigate the CpGs identified in the HCT116 colon cancer cells in primary patient samples, we analyzed hypermethylation of identified CpGs in mononuclear cells from bone marrow sample of two primary AML patients (Figure 4). Increase in DNA methylation (Δβ = 0.06, *p* = 4.68 × 10^−7^ at day 7) was observed at the identified CpGs after decitabine treatment in the bone marrow mononuclear cells of one patient (ID 1107) but not of the healthy control (median Δβ = −0.02, *p* = 0.26 at day 3), suggesting that hypermethylation in response to DNMTi might be specific to cancerous cells but not to healthy cells (Figure 4). We also observed trend of hypermethylation at identified CpGs (median Δβ = 0.06, *p* = 0.76) at day 3 in another primary leukemia patient (ID 1307) that was lost by day 14 (median Δβ = −0.01, *p* = 2.13 × 10^−35^) (Figure 4). This result again suggests that hypermethylation at identified sites after DNMTi treatment is transient also in primary leukemia cancer cells, similarly as was observed in the HCT116 cell line model. Further, significant hypermethylation was observed in 2 out of 14 PBMCs (P2 and P3) and 2 out of 6 ascites (A1 and A2) samples of platinum resistant epithelial ovarian cancer patients treated with decitabine as compared to untreated paired samples (Supplementary Figure 2). Validation of the hypermethylation at identified CpGs in the PBMCs and ascites samples treated with clinically used dose of decitabine (10 nM) further confirm our findings. We did not either observe hypermethylation in any of the eight tumor biopsy samples (Supplementary Figure 2) from the ovarian cancer patients which might be because of the requirement of higher dose of drug to result in the same effect in solid cancer as compared to PBMC and ascites samples.

### The Methylation of Identified CpGs Correlates With Expression of Corresponding Genes

**FIGURE 5:**
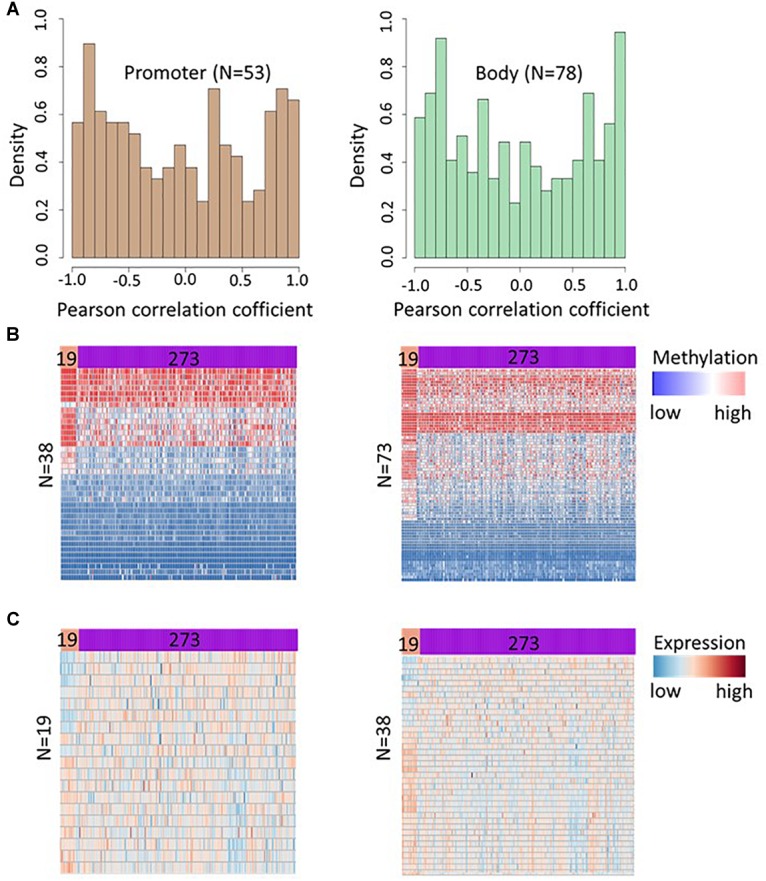
DNA methylation at hypermethylated sites affects gene expression in HCT116 cell lines and TCGA colon adenocarcinoma samples. **(A)** Histogram depicts density of Pearson correlation coefficients (PCCs) between DNA methylation and gene expression for 130 CpGs in promoter region (left panel) and 200 CpGs in gene body area (right panel). The PCCs were calculated using DNA methylation of a CpGs and the expression level of corresponding genes at day 0 (untreated cells), 5, 14, 24, and 42 days after decitabine treatment. Further, 53 promoter CpGs and 78 gene body CpGs showing a strong methylation-expression correlation (|r| ≥ 0.8) in HCT116 cell lines were selected and their methylation levels were compared between cancerous (*n* = 273) and adjacent healthy tissues (*n* = 19) samples in TCGA colon cancer data. A significant difference in methylation level (FDR < 0.05) was observed at 38 promoter and 73 gene body CpGs. **(B)** Heatmap showing the methylation level of these 38 promoters (left panel) and 73 gene body (right panel) CpGs in healthy tissue and colon cancer tissue. Subsequently, the expression level of genes corresponding to CpGs showing significant methylation differences in TCGA colon cancer data were compared and 19 genes corresponding to significant CpGs in promoter region, and 38 genes corresponding to the significant CpGs in gene body region were found differentially expressed (FDR < 0.05). **(C)** Heatmap showing the expression profile of genes with significant differences in adjacent healthy tissue (*n* = 19) and colon cancer samples (*n* = 273).

To discover the functional role of methylation in differentially methylated CpGs, we correlated DNA methylation level of identified CpGs and expression levels of the corresponding genes at multiple time points (0, 5, 14, 24, and 42 days) measured in HCT116 cell line after decitabine treatment. The distribution of correlation coefficients for 130 CpGs in promoter and 200 CpGs in gene body area has been shown in Figure 5A. A strong correlation (|r|≥ 0.80) between the gene expression and DNA methylation profiles was observed at 27% of loci (166 CpGs in 153 genes) in HCT116 cells, out of them, 47 CpGs corresponding to 44 genes were nominally significant (*p* < 0.05) (Supplementary Table 3) indicating that observed hypermethylations affect cellular function by altering gene expression in cancer cell lines. The correlation between gene expression and DNA methylation suggests that the increase in DNA methylation is functional and causes transcriptional changes that affects cellular processes (e.g., cell proliferation).

### Genes Corresponding to the Identified CpGs Show Differential Expression in Cancerous Tissue

We further investigated the pathological relevance of the increased methylation at identified CpGs using DNA methylation and gene expression profile of normal and cancerous tissue from TCGA colon adenocarcinoma patients. Out of 638 CpGs discovered in HCT116 cell line, we extensively investigated the methylation of CpGs in promoter and gene body area in TCGA data as methylations at these locations have a major effect on gene expression (Yang et al., 2014; Koch et al., 2018). We selected 53 promoter CpGs and 78 gene body CpGs showing a strong methylation-expression correlation (| r|≥ 0.8) in HCT116 cell lines and their methylation levels were compared between cancerous (*n* = 273) and adjacent healthy tissues (*n* = 19) samples in TCGA colon cancer data. This analysis revealed differential methylation of 38 promoter CpGs and 73 gene body CpGs in cancer tissue (Figure 5B and Supplementary Table 4). Subsequently, the expression level of genes corresponding to CpGs showing significant methylation differences in TCGA colon cancer data were compared and the analysis revealed significant difference (FDR < 0.05) in expression of 19 and 38 genes corresponding to differentially methylated CpGs at promoter and gene body area in colon cancer tissue respectively (Figure 5C and Supplementary Table 5). The analysis suggests that DNMTi treatment increases methylation at CpGs that involved in the pathology of cancer and hence such alteration can contribute to the therapeutic response of the drugs.

### Genes With Increased Methylation Are Enriched in Cancer-Related Pathways and Are NFAT, LEF1, and MAZ-Regulated

**FIGURE 6:**
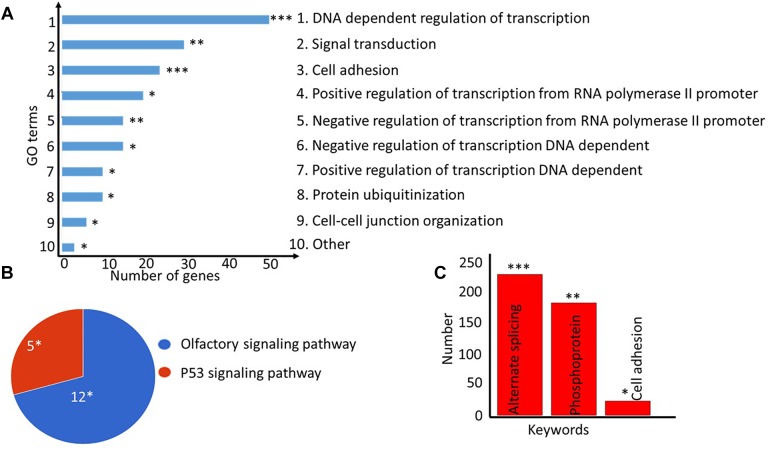
Gene ontology (GO) and pathway enrichment analyses of genes corresponding to differentially methylated CpGs. **(A)** Top-10 most significantly enriched cellular processes are shown as a bar plot. The lengths of the bars denote the number of genes present in each of the top GO categories. **(B)** Pie-chart showing the significantly enriched pathways for the genes. The number of genes present in each pathway group is shown along with the hypergeometric test *p*-value corrected for multiple testing as implemented in the DAVID tool. **(C)** The keywords enrichment analysis for the genes is shown as a bar chart. The length of the bar represents the number of genes enriched for each keyword, the FDR-corrected *p*-value is shown at the top. ^∗^FDR < 0.05; ^∗∗^FDR < 0.005; ^∗∗∗^FDR < 0.0005.

We next investigated the functions of the genes corresponding to the identified CpGs using the GeneCodis (v2) gene set enrichment analysis tool. Gene ontology enrichment analysis revealed that five out of the 10 (50%) most significant GO processes (FDR = 0.05) were related to transcription regulation, which is one of the key functional role of DNA methylation in order to control gene expression (Figure 6A). Notably, the list of enriched genes included well-known oncogenes, such as AFF3, CTNND2, ELK4, ESR1, PAX3, TRRAP, and WHSC1L1. The pathway enrichment analysis revealed that olfactory transduction and p53 signaling pathway were overrepresented (FDR = 0.05) in the gene set with increased methylation (Figure 6B), and alternating splicing was enriched as the major keyword (fold enrichment = 1.27, FDR = 0.000132, Figure 6C). Identified CpGs were also enriched in the enhancer region (*p* = 3.36 × 10^−4^) of the genomes (Supplementary Figure 3).

Furthermore, enrichment analysis among the transcription factors regulating these identified genes revealed that 59 genes were regulated by lymphoid enhancer-binding factor 1 (LEF1) (*p* = 2.1 × 10^−8^, Table 1 and Supplementary Figure 4A), 51 genes by MYC Associated Zinc Finger Protein (MAZ) (*p* = 4.07 × 10^−8^, Table 1 and Supplementary Figure 4B) and 47 of genes were regulated by the nuclear factor of activated T-cells (NFATC1, *p* = 1.48 × 10^−8^, Supplementary Figure 4C). Enrichment of genes in cancer-related pathways, especially in the p53 tumor suppressor pathway and the olfactory pathway, suggests that hypermethylation in the identified sites affects cancer cell proliferation and apoptosis.

**Table 1:** Top five transcription factor enriched in gene set corresponding to identified 638 CpGs and its regulated genes.

Transcription factor	Genes (*N*)	FDR	Regulated genes
NFATC1	47	1.48 × 10^−8^	SLIT3, RORA, SYT10, CNTNAP2, SCN3A, MRPL28, ADAMTSL1, CTNND2, ESR1, PPM1B, PDE4D, RARB, OPCML, SNX15, PRKD2, ID3, PTPRO, ADCY2, CUL3, DMD, ITM2C, KLF12, BCOR, CTNND1, SGCD, ACACA, HDAC6, POGK, AUTS2, PAX3, DLG2, SLC6A5, SOX5, DLC1, ANTXR1, NGFRAP1, LSAMP, GRM8, CACNA2D3, ETS1, S100A10, ADAMTS17, KCNH5, ARHGAP6, KCNMA1, MAP7, KCNN2
LEF1	59	2.10 × 10^−8^	PDCD10, NRXN1, SLIT3, RORA, YWHAZ, COX7B, SYNPR, SCN3A, SORCS1, TMSB4X, ADAMTSL1, NXN, CLSTN2, ZNF8, CNTN6, MAGED2, WHSC1L1, GMPR2, PDE4D, ABCF2, RARB, CNKSR2, TIA1, SMARCA1, SFRP2, OPCML, WDFY3, MBTD1, CACNA1E, PTPRO, MCTS1, DMD, KLF12, CTNND1, SGCD, ACACA, POGK, OXCT1, TAF1, PAX3, SLC6A5, SOX5, 1, SIX4, GPC6, DLC1, GTF2A2, TLE3, GAB2, TMSL3, CACNA2D3, ETS1, BZW1, ADAMTS12, CD160, TCERG1L, KCNH5, ARHGAP6, NR2F1
MAZ	51	4.07 × 10^−8^	PDCD10, SLIT3, SV2B, RORA, YWHAZ, CNTNAP2, SORCS1, PRKCI, CTNND2, ACCN2, ESR1, MAGED2, HRK, FKBP2, RARB, CNKSR2, SSR1, SMARCA1, SFRP2, PRKD2, UBE2L3, PTPRF, ID3, DMD, ITM2C, KLF12, P4HA1, BCOR, RGS7, CTNND1, DUSP6, POGK, TAF1, LYPLA2, AUTS2, DLG2, SOX5, SIX4, DLC1, TLE3, PRDM16, NGFRAP1, POLR1D, ARVCF, BZW1, THRAP3, TRRAP, ZNRF1, KCNH5, KCNMA1, NR2F1
OCT1	16	1.49 × 10^−7^	NRXN1, NR2C2, SCN3A, PDE4D, RARB, DMD, KLF12, DUSP6, SOX5, DLC1, TLE3, GAB2, TMSL3, TCERG1L, IRX2, NR2F1
MEF2	27	2.32 × 10^−7^	HIPK1, CTNNA1, NRXN3, ESR1, PPM1B, SLC12A1, SMARCA1, OPCML, WBP5, ADCY2, CUL3, DMD, KLF12, P4HA1, CTNND1, SGCD, DLG2, SOX5, GLG1, NELL2, GRM8, CACNA2D3, ETS1, CIAO1, ADAMTS12, AGTPBP1, NR2F1

*FDR-corrected p-values from the hypergeometric test are shown in the table.*

## Discussion

Our study indicates that a clinically feasible dose of decitabine (0.3 to 300 μM) treatment causes a transient increase in DNA methylation level of a small fraction of CpGs in the colon cancer genome. The use of 3-day exposure with such doses *in vitro* produces a quick increase in DNA methylation that may reflect the immediate response of cells to external stimuli. The transient increase in methylation (that vanishes 10 days after treatment) at identified targets also suggests that the response is mainly a temporary alteration of transcript level of certain genes, rather than permanent shut down or enhancement of their expression.

The further study of the hypermethylation in primary bone marrow mononuclear cells from AML patients, peripheral blood cells and ascites from epithelial ovarian cancer patients indicated that hypermethylation of subsets of the identified CpGs is an important mechanism behind DNMTi effects against primary cancer cells, which, as expected, varies across patients and cancer types. Additionally, the absence of hypermethylation in normal bone marrow mononuclear cells indicates that the hypermethylation is limited to cancerous tissues and cell lines. However, a future study exploring hypermethylation at identified CpGs in pre- and post-treated cancerous tissue types (e.g., kidney, skin, brain), in comparison to corresponding healthy cells, is needed to detail the tissue-specificity of the hypermethylation at the identified CpGs.

We did not observe hypermethylation in case of breast cancer MCF7 cell lines treated with low dose (0.06 μM) of decitabine, nor in 20 out of 26 breast cancer cell lines treated with 5 mM of azacytidine (low dose), even though the same dose of azacytidine was sufficient to hypermethylate the CpGs in colon and ovarian cancer cell lines. It suggests that lower doses of DNMTi agents are not sufficient to hypermethylate the identified CpGs in all the cancer cell lines, which could be because of their molecular peculiarities. For example, lower change in expression level of DNA methylating enzymes DNMT3A was observed in breast cancer cell lines 3 days after azacytidine treatment, as compared to colon and ovarian cancer cell lines (Supplementary Figure 5). DNMT3A is a *de novo* methylating enzyme and have been associated with CpGs hypermethylation phenotype in AML (Spencer et al., 2017). Further, the expression level of DNMT3A and DNMT3B have been associated with sensitivity to decitabine cytotoxic response in embryonic cells (Oka et al., 2005), testicular germ cell tumor (Beyrouthy et al., 2009) and have also shown to be predictive of decitabine treatment response in patient-derived xenograft model for triple negative breast cancer (Yu et al., 2018). Differences in expression of DNMT enzymes, together other molecular differences (e.g., genetic mutations or epigenetic differences) may resists breast cancer cells against the hypermethylation in response to DNMTi at a lower dose. However, a detailed mechanistic study will be needed to understand the absence of hypermethylation in breast cancer cells in relation with DNMT3A or DNMT3B levels and other molecular heterogeneity.

Additionally, genes associated with identified CpGs were enriched among olfactory receptor and p53 pathways. P53 pathway regulates DNA repair and apoptosis in HCT116 cells (Li et al., 2015), and there is growing evidence for the involvement of olfactory receptor pathway in cancers of non-olfactory tissues such as prostate (Neuhaus et al., 2009), lung (Giandomenico et al., 2013; Kalbe et al., 2017), and colon cancers (Morita et al., 2016). For instance, olfactory receptor OR51B4 is highly expressed in colon cancer HCT116 cells and the activation of the receptor by its ligand (Troenan) inhibited cell proliferation and apoptosis by phosphorylation of p38, mTOR, and Akt kinases (Weber et al., 2017). The available literature therefore suggests that identified hypermethylation can affect colon cancer cells proliferation and apoptosis through olfactory receptor pathway. However, a detailed mechanistic study is warranted to decipher the exact role of hypermethylation at identified CpGs in cellular growth through p53 and olfactory receptor pathways.

Further, enriched transcription factor LEF1 is implicated in tumorigenesis and cancer cell proliferation, migration, invasion, and stemness in multiple cancer (e.g., colorectal cancer, AML, oral squamous cell carcinoma) (Santiago et al., 2017). Moreover, the methylation at a fraction of CpGs was positively correlated with the population doubling time of HCT116 cells indicating the role of identified CpGs in cell division, and proliferation. Enrichment of genes among the cancer-related pathways involved in cell proliferation and apoptosis suggests a non-random selection of CpGs for hypermethylation in order to alter the cellular dynamics (e.g., population doubling time). We consider that increased in methylation at identified CpGs can contribute to the drug response in multiple cancer types partly by altering the cell division rate. The methylation levels at identified CpGs could be useful to design better treatment strategies (e.g., predicting the DNMTi responders and non-responders), and to understand side effects due to higher drug dose (e.g., the increase in DNA methylation at identified CpGs could be an indicator for degree of possible toxic effect). These novel findings therefore warrant further analyses in a number of cancer types using either pre-clinical animal studies or clinical treatment data from cancer patients with baseline molecular profiles available.

There are multiple reasons to suggest that at least one key mechanism underlying the anti-tumor responses to DNMTi treatment may involve hypermethylation of specific genes in cancer cells. First, we showed an increase in DNA methylation at identified CpGs in more than one type of cancer cells. Second, as defined for an epigenetic change, these sustained changes persist for significant periods of time (at least more than 5 days) after a transient, subsequently withdrawn, drug exposure (in this case 72 h). Third, the expression patterns for a subset of the genes are different between cancer and normal tissue types. Importantly, these changes are induced by drug doses that do not acutely kill cells and, thus, allow the transient alterations in gene methylation patterns to act on emerging molecular changes to cells after DNMTi therapy.

In summary, our findings suggest that DNMTi mediates its therapeutic effect through a complex mechanism of action, and therefore a generalized pattern for the activity is challenging to find as DNMTi treatment causes hypermethylation at certain loci and hypomethylation at others. Hence, the effects of DNMTi on cancer tissues should be analyzed at the individual gene level, rather than at the entire genomic level, and separately for each tissue type and even for each cancer patient. Further, any attempt to predict DNMTi response should also incorporate the hypermethylated targets of the drugs along with the hypomethylated targets of the drugs. However, a functional mechanistic study in more advanced model systems and several human tissue types will be required for further revealing how the increase in methylation at identified loci alters treatment response and the pathological burden of disease. We hope that these identified targets associated with hypermethylation have broad implications for further research on DNMTi response mechanisms in multiple cancer types.

## Author Contributions

AG conceptualized, analyzed the data, and wrote the manuscript. TA critically revised and edited the manuscript.

## Conflict of Interest Statement

The authors declare that the research was conducted in the absence of any commercial or financial relationships that could be construed as a potential conflict of interest.

## Footnotes

1 http://gdac.broadinstitute.org/

2 ftp://ussd-ftp.illumina.com/downloads/ProductFiles/HumanMethylation450/HumanMethylation450_15017482_v1-2.csv
