# Supplementary material for: DNMT Inhibitors Increase Methylation in the Cancer Genome
Source: Front Pharmacol. 2019 Apr 24;10:385. doi: 10.3389/fphar.2019.00385 (PMC6491738; doi:10.3389/fphar.2019.00385)
Supplement: Supplementary file 1 [file Data_Sheet_1.pdf]

Supplementary Table 1. List of 638 CpG showing increased methylation after decitabine treatment in HCTK116 cell line

Chromosome	Base position	CpG	Location in gene	Gene
5	76332945	ch.5.1443044F	Promoter	AGGF1
10	79793495	cg23620279	Promoter	RPS24
12	58087540	cg18202167	Promoter	OS9
12	58087544	cg00062356	Promoter	OS9
7	72299837	cg19955956	Promoter	SBDSP
2	231729487	cg15127563	Promoter	ITM2C
15	70390363	cg07020846	Promoter	TLE3
6	33282175	cg06097707	Promoter	TAPBP
8	17942583	cg07312099	Promoter	ASAH1
X	53310991	cg14972383	Promoter	IQSEC2
7	150924351	cg10436877	Promoter	ABCF2
X	12993075	cg23376554	Promoter	TMSB4X
X	102510216	cg14004049	Promoter	TCEAL8
1	43996436	cg14172596	Promoter	PTPRF
1	113933413	cg18539474	Promoter	MAGI3
7	107221074	cg11785538	Promoter	BCAP29
13	49684397	cg17091793	Promoter	FNDC3A
5	78281964	cg26802063	Promoter	ARSB
X	146312384	cg27167381	Promoter	MIR506
X	77154874	cg10646076	Promoter	COX7B
4	7105115	cg26600181	Promoter	FLJ36777
10	91152122	cg16395953	Promoter	IFIT1
X	101186742	cg23922730	Promoter	ZMAT1
4	47033180	cg21472546	Promoter	GABRB1
1	1981816	cg22865720	Promoter	PRKCZ
11	55587104	cg08060810	Promoter	OR5D18
10	63661280	cg16253809	Promoter	ARID5B
1	23886472	cg20485144	Promoter	ID3
19	58790298	cg23548487	Promoter	ZNF8
X	145082826	cg15918587	Promoter	MIR891B
19	13885098	cg09952620	Promoter	C19orf53
11	5905350	cg06484232	Promoter	OR52E4
13	103426305	cg13675958	Promoter	C13orf27
16	5008134	cg08043782	Promoter	SEC14L5
5	158634905	cg26362852	Promoter	RNF145
19	20368371	cg22155405	Promoter	LOC284441
19	4066818	cg10561472	Promoter	ZBTB7A
6	13615538	cg05884522	Promoter	NOL7

2	39005241	cg15934678	Promoter	GEMIN6
5	92918848	cg05945291	Promoter	NR2F1
X	2984799	cg17012513	Promoter	ARSF
10	15210836	cg23193446	Promoter	NMT2
19	20011538	cg27379065	Promoter	ZNF93
13	28194831	cg07375367	Promoter	POLR1D
1	225616668	cg20483690	Promoter	LBR
X	119737675	cg05782751	Promoter	MCTS1
19	54694174	cg12173535	Promoter	MBOAT7
19	37020332	cg24909706	Promoter	ZNF260
11	131779469	cg08097520	Promoter	NTM
15	72524656	cg25016070	Promoter	PKM2
4	25162716	cg19113954	Promoter	SEPSECS
5	92918072	cg16448525	Promoter	FLJ42709
6	160211006	cg07151830	Promoter	TCP1
5	135702333	cg16219583	Promoter	TRPC7
5	135701422	cg17275074	Promoter	TRPC7
8	24240597	cg14143055	Promoter	ADAMDEC1
1	3606550	cg21388339	Promoter	TP73
X	153169465	cg20664654	Promoter	AVPR2
8	28748404	cg06241765	Promoter	INTS9
X	102629870	cg13486082	Promoter	NGFRAP1
5	92917334	cg08003613	Promoter	FLJ42709
2	70476188	cg24074685	Promoter	TIA1
X	102629912	cg25198830	Promoter	NGFRAP1
X	12992684	cg17625764	Promoter	TMSL3
19	30432806	cg27180365	Promoter	C19orf2
12	50450889	cg23126949	Promoter	ACCN2
13	34391699	cg08155354	Promoter	RFC3
14	75535757	cg15979150	Promoter	FAM164C
3	69129729	cg21635584	Promoter	UBA3
1	25559743	cg17651255	Promoter	SYF2
9	113800909	cg06148685	Promoter	LPAR1
12	19282261	cg13108328	Promoter	PLEKHA5
18	48404491	cg26727372	Promoter	ME2
X	128657727	cg18959966	Promoter	SMARCA1
1	10532838	cg21149582	Promoter	DFFA
X	15354150	cg18016370	Promoter	PIGA
4	85888001	cg14553853	Promoter	WDFY3
11	108092818	cg12019961	Promoter	ATM
20	20032580	cg06599170	Promoter	C20orf26
4	68567439	cg25695041	Promoter	UBA6
3	52719268	cg09817993	Promoter	GNL3
6	30655567	cg23903723	Promoter	KIAA1949
9	139376822	cg13820039	Promoter	C9orf163

11	79114133	cg25837979	Promoter	MIR708
5	149379518	cg26588194	Promoter	HMGXB3
1	145469887	cg12222699	Promoter	ANKRD34A
6	3458177	cg11713788	Promoter	SLC22A23
19	12625436	cg24109012	Promoter	ZNF709
13	29597447	cg07790085	Promoter	MTUS2
11	5019849	cg23434090	Promoter	OR51L1
14	61191253	cg09970023	Promoter	SIX4
1	234040045	cg10878114	Promoter	SLC35F3
5	54319373	cg14597388	Promoter	GZMK
11	51413644	cg23935054	Promoter	OR4A5
16	420755	cg09504571	Promoter	MRPL28
19	20749738	cg12124647	Promoter	ZNF737
10	13628544	cg15881990	Promoter	PRPF18
10	15902872	cg19707359	Promoter	FAM188A
1	152087267	cg22603037	Promoter	TCHH
5	140592867	cg14640659	Promoter	PCDHB13
11	111807548	cg11471799	Promoter	DIXDC1
11	128458153	cg16792062	Promoter	ETS1
11	55577775	cg05788138	Promoter	OR5L1
14	78869352	cg10828316	Promoter	NRXN3
12	113773298	cg12583184	Promoter	SLC24A6
X	77151316	cg11290168	Promoter	MAGT1
15	102344469	cg26155802	Promoter	OR4F6
X	154254823	cg09526164	Promoter	FUNDC2
1	16010601	cg09073052	Promoter	PLEKHM2
11	55796572	cg17060964	Promoter	OR5AS1
15	54303927	cg22845496	Promoter	UNC13C
11	123814972	cg15625631	Promoter	OR6T1
4	184365198	cg24787081	Promoter	CDKN2AIP
1	159258877	cg14696870	Promoter	FCER1A
19	14683110	cg16256643	Promoter	NDUFB7
22	20004611	cg12912949	Promoter	ARVCF
1	151967449	cg06698332	Promoter	S100A10
X	99892000	cg11509733	Promoter	TSPAN6
14	70715730	cg20576094	Promoter	ADAM21P1
1	241694605	cg11150901	Promoter	KMO
5	68513219	cg15387943	Promoter	MRPS36
5	159846543	cg15333689	Promoter	SLU7
14	55595666	cg26335127	Promoter	LGALS3
X	77154732	cg24112882	Promoter	COX7B
4	155471778	cg11404039	Promoter	PLRG1
6	52149972	cg18225895	Promoter	MCM3
12	117319577	cg14276619	Promoter	HRK
10	44287023	cg17952824	Promoter	HNRNPA3P1

12	67662516	cg18750937	Promoter	CAND1
1	248568331	cg13053563	Promoter	OR2T1
17	3627058	cg14971744	Body	ITGAE
15	89010209	cg20630605	Body	MRPL46
12	97885270	cg27533635	Body	RMST
X	131547702	cg13633856	Body	MBNL3
X	131547697	cg14520512	Body	MBNL3
8	12974556	cg06103928	Body	DLC1
4	76439140	cg12093136	Body	RCHY1
5	54455564	cg08212230	Body	CDC20B
21	39494547	cg25816610	Body	DSCR8
1	231820076	cg07134368	Body	TSNAX-DISC1
1	231964048	cg22367981	Body	DISC1
1	44287964	cg23290313	Body	ST3GAL3
3	171138553	cg22901347	Body	TNIK
2	223151884	cg11490745	Body	PAX3
16	74565916	ch.16.1684049R	Body	GLG1
12	45034784	ch.12.897509F	Body	NELL2
19	52862167	cg10341573	Body	ZNF610
1	114503218	ch.1.2681285F	Body	HIPK1
10	79033545	cg08772567	Body	KCNMA1
14	33826344	cg15454195	Body	NPAS3
19	53619086	cg22834281	Body	ZNF415
X	21559778	ch.X.346519R	Body	CNKSR2
20	45937282	ch.20.1002962F	Body	ZMYND8
7	69447465	cg09703727	Body	AUTS2
5	155909246	cg24132325	Body	SGCD
2	205591269	cg18626478	Body	PARD3B
X	70661061	ch.X.1084407R	Body	TAF1
10	83848597	cg17519477	Body	NRG3
3	189353419	cg25708695	Body	TP63
22	21968010	ch.22.149158R	Body	UBE2L3
14	80324276	cg19753609	Body	NRXN3
7	69478390	cg16819888	Body	AUTS2
7	154006066	cg07467482	Body	DPP6
2	165998136	cg16631432	Body	SCN3A
5	156097036	cg15160274	Body	SGCD
2	50570407	cg06707406	Body	NRXN1
20	34297200	ch.20.707667F	Body	RBM39
7	147709862	cg22807241	Body	MIR548F3
11	115369647	cg11019127	Body	CADM1
3	25635650	cg07405178	Body	RARB
10	108674143	cg23024358	Body	SORCS1
4	85766242	ch.4.1647744F	Body	WDFY3
2	100371023	cg22092126	Body	AFF3

2	153575717	cg07491444	Body	ARL6IP6
13	34392781	cg24492140	Body	RFC3
2	159173778	cg21514997	Body	CCDC148
12	23998997	cg06764736	Body	SOX5
13	43545157	cg17400905	Body	EPSTI1
11	115096810	cg25461513	Body	CADM1
19	19923956	ch.19.841535R	Body	ZNF506
1	28886386	ch.1.953398R	Body	TRNAU1AP
2	116480197	cg06394103	Body	DPP10
5	59126518	cg27583655	Body	PDE4D
22	46114168	ch.22.909671F	Body	ATXN10
1	16715418	ch.1.572291F	Body	C1orf144
5	113805552	cg25486361	Body	KCNN2
13	60543691	ch.13.865492R	Body	DIAPH3
2	100175805	cg17165836	Body	AFF3
2	100365075	cg13361307	Body	AFF3
15	76136846	cg16242106	Body	UBE2Q2
12	50635579	ch.12.1023240F	Body	LIMA1
2	227850069	cg09157320	Body	RHBDD1
20	10026325	ch.20.221631R	Body	ANKRD5
11	64795449	cg21821990	Body	SNX15
12	117611422	ch.12.2406115F	Body	FBXO21
14	63508497	cg25609301	Body	KCNH5
8	38174205	ch.8.903080R	Body	WHSC1L1
2	192543258	cg15794798	Body	OBFC2A
7	150452495	cg14319487	Body	LOC100128542
15	61346347	cg08099431	Body	RORA
15	24411878	cg15564871	Body	PWRN2
10	132942686	cg06938601	Body	TCERG1L
13	26483520	cg12565580	Body	ATP8A2
3	194136354	ch.3.3822654R	Body	ATP13A3
5	11529629	cg16051561	Body	CTNND2
6	1836850	cg21478123	Body	GMDS
16	75044269	ch.16.1700675R	Body	ZNRF1
10	132942731	cg25486749	Body	TCERG1L
5	166938213	cg23167425	Body	ODZ2
11	92264986	cg07276831	Body	FAT3
18	34194679	ch.18.672159R	Body	FHOD3
16	9010914	ch.16.350833F	Body	USP7
3	130299763	cg17937340	Body	COL6A6
6	1909853	cg11276500	Body	GMDS
10	15901893	cg19053479	Body	FAM188A
7	48319696	cg10626169	Body	ABCA13
1	45187551	cg07722722	Body	C1orf228
3	140229290	cg23463099	Body	CLSTN2

10	96306185	cg10069677	Body	HELLS
11	122037845	cg15826891	Body	LOC399959
5	168395173	cg11458498	Body	SLIT3
21	34978286	cg12708807	Body	CRYZL1
5	171653553	ch.5.3268483F	Body	UBTD2
15	80188894	cg07293993	Body	MTHFS
12	50527085	ch.12.1019410F	Body	LASS5
1	10464086	ch.1.385573R	Body	PGD
3	21558209	cg16439360	Body	ZNF385D
1	241474238	cg08238568	Body	RGS7
5	138146102	ch.5.2559743R	Body	CTNNA1
5	41837108	ch.5.884579R	Body	OXCT1
10	114074843	cg18014500	Body	GUCY2G
1	181514216	cg22359828	Body	CACNA1E
17	1717862	ch.17.79071R	Body	SMYD4
5	7686199	cg16253976	Body	ADCY2
2	225441832	cg11229715	Body	CUL3
14	91751397	ch.14.1452150F	Body	CCDC88C
8	35401908	cg22872195	Body	UNC5D
8	97855859	cg08247527	Body	PGCP
10	34817409	cg19017553	Body	PARD3
10	106749483	cg26349484	Body	SORCS3
3	35730993	cg15459537	Body	ARPP-21
9	14838752	cg13762569	Body	FREM1
15	41734226	ch.15.433532F	Body	RTF1
6	7297596	ch.6.197209F	Body	SSR1
13	94493055	cg21222888	Body	GPC6
8	119282796	ch.8.2353618R	Body	SAMD12
5	15780558	ch.5.409282R	Body	FBXL7
4	72635202	cg24806812	Body	GC
2	211401919	ch.2.4215183F	Body	CPS1
3	64801329	cg21324884	Body	MIR548A2
4	110553306	ch.4.2065340F	Body	CCDC109B
2	105686129	ch.2.2207852R	Body	MRPS9
1	212238115	ch.1.4129519F	Body	DTL
6	12910610	cg14773588	Body	PHACTR1
5	118874673	ch.5.2173511R	Body	HSD17B4
7	150451091	cg06276978	Body	LOC100128542
20	29847402	cg25361651	Body	DEFB115
3	54807076	cg19093405	Body	CACNA2D3
1	206768238	ch.1.4018176R	Body	LGTN
1	247691111	cg25139877	Body	LOC148824
4	44425078	cg12709692	Body	KCTD8
3	45553017	cg15691035	Body	LARS2
13	74491964	ch.13.1085822R	Body	KLF12

7	148111040	ch.7.3089487R	Body	CNTNAP2
3	121977827	cg10364968	Body	CASR
6	167200499	cg18495191	Body	RPS6KA2
17	49281558	ch.17.1348593F	Body	MBTD1
1	21074008	ch.1.705736F	Body	HP1BP3
18	55024674	cg21518865	Body	ST8SIA3
5	33698653	ch.5.731560F	Body	ADAMTS12
10	69913749	cg18986048	Body	MYPN
2	1182847	ch.2.35699F	Body	SNTG2
8	144798631	cg22892110	Body	MAPK15
15	48515109	cg23530596	Body	SLC12A1
6	33393183	cg11261678	Body	SYNGAP1
1	36766063	ch.1.1168472R	Body	THRAP3
6	33395430	cg19968421	Body	SYNGAP1
5	136682394	cg19567866	Body	SPOCK1
6	136828807	ch.6.2623783F	Body	MAP7
9	18825658	cg13724111	Body	ADAMTSL1
3	124726246	ch.3.2442921F	Body	HEG1
4	154707153	cg11467638	Body	SFRP2
3	51637060	ch.3.1119246R	Body	RAD54L2
5	160053412	cg27141889	Body	ATP10B
8	14108012	ch.8.362960F	Body	SGCZ
3	73591147	cg09236445	Body	PDZRN3
X	154255950	cg10432310	Body	FUNDC2
20	51648147	cg09566894	Body	TSHZ2
4	159918056	cg22266824	Body	C4orf45
2	44182106	ch.2.1056241F	Body	LRPPRC
9	111807413	ch.9.1678974F	Body	C9orf5
20	24953309	ch.20.532344R	Body	C20orf3
17	48194635	cg11441693	Body	SAMD14
14	79558823	cg18818949	Body	NRXN3
12	122065180	cg24082347	Body	ORAI1
5	98107521	cg12949466	Body	RGMB
1	241176676	cg22231602	Body	RGS7
19	12277357	cg13689563	Body	ZNF136
20	62645068	ch.20.1534602F	Body	PRPF6
15	61500965	cg20124735	Body	RORA
15	59940044	ch.15.825727F	Body	GTF2A2
8	13092547	ch.8.343778F	Body	DLC1
5	126871056	ch.5.2320326F	Body	PRRC1
11	47659584	ch.11.997072R	Body	MTCH2
11	20621341	cg20632573	Body	SLC6A5
17	837017	cg07494499	Body	NXN
4	121668750	ch.4.2245532F	Body	PRDM5
19	10401361	cg07097925	Body	ICAM5

3	169994002	ch.3.3303606F	Body	PRKCI
1	242310145	cg07977614	Body	PLD5
5	167028535	cg15651267	Body	ODZ2
3	15058168	ch.3.343413R	Body	NR2C2
8	36763165	cg12033248	Body	KCNU1
11	132931732	cg18413062	Body	OPCML
7	32038956	cg13298997	Body	PDE1C
9	88291443	ch.9.1152820R	Body	AGTPBP1
X	10087726	cg25497053	Body	WWC3
12	126055778	cg19992906	Body	TMEM132B
7	98536084	ch.7.2068158F	Body	TRRAP
4	128654094	cg13665890	Body	SLC25A31
7	79765394	cg25702790	Body	GNAI1
11	78071309	ch.11.1702122F	Body	GAB2
12	33589594	cg06721860	Body	SYT10
11	63140804	cg10116443	Body	SLC22A9
8	126142264	cg12803053	Body	NSMCE2
3	159590447	cg09811510	Body	SCHIP1
3	1382597	cg16522250	Body	CNTN6
3	115618688	cg16752940	Body	LSAMP
4	6676521	cg16758887	Body	LOC93622
11	78400028	cg07441953	Body	ODZ4
12	42877995	cg20908919	Other	PRICKLE1
4	130017238	cg13107060	Other	C4orf33
19	13262082	cg15340644	Other	IER2
5	59782121	cg18611813	Other	PDE4D
21	37433149	cg17039262	Other	SETD4
12	88536565	cg20328917	Other	TMTC3
12	121790694	cg12210527	Other	ANAPC5
6	36409495	cg06422757	Other	PXT1
1	34328907	cg05723953	Other	HMGB4
X	31889692	cg20522855	Other	DMD
11	83393062	cg13572369	Other	DLG2
19	37061383	cg10172250	Other	ZNF529
X	11282604	cg08456555	Other	ARHGAP6
6	153303350	cg18198306	Other	FBXO5
6	31515398	cg00002930	Other	NFKBIL1
X	54834954	cg09208571	Other	MAGED2
20	47778018	ch.20.1062061F	Other	STAU1
8	101963358	cg11839355	Other	YWHAZ
10	43902500	cg12891252	Other	HNRNPF
10	74855378	cg19832312	Other	P4HA1
X	39954231	cg07099245	Other	BCOR
18	3263082	cg19146448	Other	MYL12B
19	47219957	cg25522119	Other	PRKD2

9	74979581	cg14026485	Other	ZFAND5
2	208489667	cg07496861	Other	FAM119A
18	13375474	cg26700919	Other	C18orf1
3	132772692	cg24837219	Other	TMEM108
2	159905508	ch.2.3260358R	Other	TANC1
4	129731973	cg11264547	Other	PHF17
18	13375540	cg21243597	Other	C18orf1
3	114599007	cg19149693	Other	ZBTB20
15	91646263	cg27121538	Other	SV2B
X	50028082	cg11113650	Other	CCNB3
1	205599988	cg15558299	Other	ELK4
18	13229005	ch.18.316502R	Other	C18orf1
11	57529465	cg19210276	Other	CTNND1
22	29137759	cg22585269	Other	CHEK2
X	150867017	cg15731296	Other	PRRG3
19	38827331	cg09094448	Other	CATSPERG
6	152085641	cg18132851	Other	ESR1
X	101186679	cg26142661	Other	ZMAT1
12	24577904	cg24011341	Other	SOX5
1	26565342	ch.1.876374R	Other	CCDC21
5	147101975	cg11799006	Other	JAKMIP2
10	21807252	cg25195795	Other	C10orf140
2	16839610	cg09324018	Other	FAM49A
3	125093863	cg15705999	Other	ZNF148
3	176914208	cg07883762	Other	TBL1XR1
16	62067937	cg07244927	Other	CDH8
6	166581272	cg00070318	Other	T
1	158149974	cg24432768	Other	CD1D
4	87857667	cg19533294	Other	AFF1
12	87106229	cg14783993	Other	MGAT4C
11	85359560	cg26796873	Other	TMEM126A
1	33722623	cg11416597	Other	ZNF362
18	21976748	cg20528338	Other	OSBPL1A
2	64880293	cg11920737	Other	SERTAD2
X	48660813	cg10783042	Other	HDAC6
1	24118400	cg11659749	Other	LYPLA2
2	201677426	cg14211387	Other	BZW1
7	32526065	cg26856631	Other	LSM5
19	57324295	cg08155759	Other	PEG3
19	37001658	cg25505109	Other	ZNF260
15	100253379	ch.15.1787851R	Other	MEF2A
5	2746667	cg07766803	Other	IRX2
19	21608124	cg19023258	Other	ZNF493
3	63602009	cg11876912	Other	SYNPR
19	12299253	cg21880712	Other	ZNF136

15	20737822	cg14783259	Other	GOLGA6L6
19	53642858	cg15050103	Other	ZNF347
18	8638712	ch.18.189560F	Other	RAB12
19	20231820	cg06736434	Other	ZNF90
2	44460827	cg19769080	Other	PPM1B
10	78635553	cg23533270	Other	KCNMA1
7	117835958	cg13799581	Other	NAA38
6	25788879	cg06885175	Other	SLC17A1
10	32300362	ch.10.820670F	Other	KIF5B
X	7270088	cg10073470	Other	STS
4	6643382	cg20272423	Other	MRFAP1
2	96939558	ch.2.2007613R	Other	CIAO1
15	30930499	cg00067141	Other	ARHGAP11B
14	24701654	cg07519822	Other	GMPR2
15	99789637	cg25385940	Other	TTC23
X	80457315	cg21896142	Other	HMGN5
18	616707	cg23661343	Other	CLUL1
X	102611415	cg27464574	Other	WBP5
X	102611412	cg13208102	Other	WBP5
10	81107244	cg16098780	Other	PPIF
1	85528044	cg22488158	Other	WDR63
X	99899378	cg24666876	Other	SRPX2
X	77154996	cg15830530	Other	COX7B
20	43835661	cg11264863	Other	SEMG1
4	25235765	cg12931625	Other	PI4K2B
8	65496126	cg07205627	Other	BHLHE22
12	62654245	cg18915437	Other	USP15
8	145735102	cg17958180	Other	MFSD3
11	55606710	cg12962308	Other	OR5D16
6	32634362	cg05724777	Other	HLA-DQB1
3	149057820	cg06305422	Other	Intergeneic
3	152871396	ch.3.3016567F	Other	Intergeneic
2	19616327	cg21039221	Other	Intergeneic
1	209365350	cg22736624	Other	Intergeneic
3	116936427	cg11607648	Other	Intergeneic
8	89664399	ch.8.89733515F	Other	Intergeneic
22	32046895	ch.22.436090R	Other	Intergeneic
11	105098067	ch.11.104603277F	Other	Intergeneic
5	123635353	ch.5.2251785F	Other	Intergeneic
13	66774231	ch.13.65672232R	Other	Intergeneic
1	11967826	cg22340067	Other	Intergeneic
15	53205349	ch.15.50992641R	Other	Intergeneic
2	129663717	cg19404692	Other	Intergeneic
10	44099144	cg26270975	Other	Intergeneic
4	162116791	ch.4.162336241R	Other	Intergeneic

16	49903138	ch.16.48460639F	Other	Intergeneic
2	81088536	ch.2.80942047R	Other	Intergeneic
6	26595126	cg19497998	Other	Intergeneic
2	8397810	cg19256423	Other	Intergeneic
14	86554255	cg13090238	Other	Intergeneic
8	37006314	ch.8.870369R	Other	Intergeneic
6	128891095	cg18500322	Other	Intergeneic
1	161339556	ch.1.159606180R	Other	Intergeneic
12	85844287	ch.12.1700408R	Other	Intergeneic
8	54188341	ch.8.54350894R	Other	Intergeneic
14	53850070	ch.14.628538R	Other	Intergeneic
9	76690975	ch.9.919537F	Other	Intergeneic
2	227865079	ch.2.4543734F	Other	Intergeneic
11	81757203	ch.11.1767550R	Other	Intergeneic
18	5233979	cg26881207	Other	Intergeneic
X	33740873	cg07983986	Other	Intergeneic
2	205130274	cg17591195	Other	Intergeneic
8	119971687	cg21022303	Other	Intergeneic
2	118979739	cg27358426	Other	Intergeneic
11	15363270	cg09663343	Other	Intergeneic
8	2483325	cg17224775	Other	Intergeneic
5	178483871	cg11282433	Other	Intergeneic
2	157192128	cg12335829	Other	Intergeneic
3	137492929	cg15062059	Other	Intergeneic
3	16768672	ch.3.382096F	Other	Intergeneic
10	132834807	cg11315633	Other	Intergeneic
2	9188970	ch.2.246819F	Other	Intergeneic
15	75206153	ch.15.72993206F	Other	Intergeneic
3	71636085	cg06479142	Other	Intergeneic
14	54572859	cg24011936	Other	Intergeneic
6	164526833	cg21567971	Other	Intergeneic
2	181200445	ch.2.180908690F	Other	Intergeneic
16	13461010	ch.16.486323F	Other	Intergeneic
8	26942425	cg23760300	Other	Intergeneic
6	163768411	cg20867674	Other	Intergeneic
5	60527978	ch.5.1161320F	Other	Intergeneic
13	54817511	cg09602751	Other	Intergeneic
9	97015266	ch.9.96055087R	Other	Intergeneic
2	5374307	ch.2.154144R	Other	Intergeneic
20	11513716	ch.20.250771F	Other	Intergeneic
5	169008308	cg09106932	Other	Intergeneic
10	79401752	cg27024057	Other	Intergeneic
3	179993335	cg22289155	Other	Intergeneic
12	46950853	cg23677778	Other	Intergeneic
6	27243037	cg21643086	Other	Intergeneic

15	47565787	cg10477878	Other	Intergeneic
13	87224101	cg21275368	Other	Intergeneic
19	37892209	cg26361327	Other	Intergeneic
18	5888060	cg06977186	Other	Intergeneic
17	39010551	cg07958689	Other	Intergeneic
6	24975742	ch.6.25083721F	Other	Intergeneic
2	14336631	cg12676991	Other	Intergeneic
6	26330589	cg13569146	Other	Intergeneic
11	34608061	cg22314759	Other	Intergeneic
5	3779072	cg18924848	Other	Intergeneic
16	65800351	ch.16.1425090F	Other	Intergeneic
5	180097910	cg23214352	Other	Intergeneic
14	63131938	cg20468787	Other	Intergeneic
7	112135961	cg23222472	Other	Intergeneic
10	86910486	cg19088503	Other	Intergeneic
6	94550257	ch.6.94606978F	Other	Intergeneic
14	88608773	cg13997469	Other	Intergeneic
7	125664997	ch.7.125452233F	Other	Intergeneic
3	151555029	cg21509105	Other	Intergeneic
1	2689171	cg21584800	Other	Intergeneic
7	69058543	cg12999084	Other	Intergeneic
2	2757161	cg18663259	Other	Intergeneic
5	3187456	cg05712938	Other	Intergeneic
15	35405377	ch.15.33192669F	Other	Intergeneic
7	51658747	cg12988117	Other	Intergeneic
19	42439146	cg16700658	Other	Intergeneic
2	173575577	cg08641935	Other	Intergeneic
2	124194392	ch.2.123910862R	Other	Intergeneic
2	227291401	cg25101764	Other	Intergeneic
5	56790874	cg06821992	Other	Intergeneic
6	164614646	cg10413861	Other	Intergeneic
5	1742623	cg12501402	Other	Intergeneic
X	124332875	cg09740875	Other	Intergeneic
3	59501481	cg06146466	Other	Intergeneic
3	177416888	ch.3.3451078R	Other	Intergeneic
6	78002283	cg13663057	Other	Intergeneic
2	227342994	cg13165983	Other	Intergeneic
14	98097986	cg10319905	Other	Intergeneic
11	49073835	cg06445586	Other	Intergeneic
17	32633974	cg12243622	Other	Intergeneic
13	55812567	ch.13.54710568F	Other	Intergeneic
3	16779726	cg27614376	Other	Intergeneic
12	97951712	cg27109238	Other	Intergeneic
16	52641824	cg10109421	Other	Intergeneic
13	91343397	ch.13.90141398F	Other	Intergeneic

13	35200731	ch.13.381084F	Other	Intergeneic
10	62576479	cg09868354	Other	Intergeneic
X	138525808	cg09148853	Other	Intergeneic
3	8041501	cg16361249	Other	Intergeneic
10	93412294	cg16377790	Other	Intergeneic
1	88167694	ch.1.87940282F	Other	Intergeneic
21	25582901	cg25946965	Other	Intergeneic
7	136322567	cg22918741	Other	Intergeneic
10	54203646	cg23803709	Other	Intergeneic
7	77269758	ch.7.1700983F	Other	Intergeneic
5	72750474	cg14102740	Other	Intergeneic
13	31972778	ch.13.315182R	Other	Intergeneic
11	34608041	cg16935203	Other	Intergeneic
4	24274965	cg15650745	Other	Intergeneic
8	145910623	cg24497813	Other	Intergeneic
X	136921659	cg17550929	Other	Intergeneic
21	30188220	ch.21.284298R	Other	Intergeneic
6	67606015	ch.6.1433484F	Other	Intergeneic
16	51475605	cg27268835	Other	Intergeneic
14	86687661	cg07450805	Other	Intergeneic
5	158534530	cg19070856	Other	Intergeneic
4	19777808	cg24770408	Other	Intergeneic
1	171407789	cg25061682	Other	Intergeneic
3	44895117	ch.3.44870121R	Other	Intergeneic
12	116784790	cg16326611	Other	Intergeneic
13	56790593	cg23817132	Other	Intergeneic
14	63112650	cg11159234	Other	Intergeneic
3	70048377	cg06341047	Other	Intergeneic
2	30146945	cg07060894	Other	Intergeneic
17	68663564	ch.17.66175159R	Other	Intergeneic
10	10462173	ch.10.290763R	Other	Intergeneic
2	166937869	cg13875008	Other	Intergeneic
16	3201981	cg06643150	Other	Intergeneic
17	37309414	cg10213328	Other	Intergeneic
12	65174660	cg14078059	Other	Intergeneic
3	75862225	ch.3.1652793F	Other	Intergeneic
3	19740602	cg07849811	Other	Intergeneic
X	150864703	cg09026179	Other	Intergeneic
4	24043352	cg25659893	Other	Intergeneic
1	214435182	cg16682225	Other	Intergeneic
11	112693829	cg24986840	Other	Intergeneic
2	5689033	cg13362028	Other	Intergeneic
6	166270367	cg16206344	Other	Intergeneic
15	26328941	cg23527974	Other	Intergeneic
20	52793130	cg25305530	Other	Intergeneic

3	88930955	ch.3.89013645F	Other	Intergeneic
10	32254360	ch.10.819441R	Other	Intergeneic
2	8416612	ch.2.224494R	Other	Intergeneic
13	47994694	cg18423626	Other	Intergeneic
11	23248542	ch.11.535384R	Other	Intergeneic
1	89027289	ch.1.88799877F	Other	Intergeneic
3	117075024	cg12837919	Other	Intergeneic
19	12904162	cg15317793	Other	Intergeneic
3	175640408	ch.3.3414728R	Other	Intergeneic
X	141126129	ch.X.2058079F	Other	Intergeneic
4	125375437	ch.4.125594887R	Other	Intergeneic
X	34404521	cg17001761	Other	Intergeneic
12	66119400	cg18758900	Other	Intergeneic
2	103593390	cg15128147	Other	Intergeneic
1	14477617	cg23803120	Other	Intergeneic
6	14426636	ch.6.14534615F	Other	Intergeneic
2	21874939	cg16784006	Other	Intergeneic
2	240866924	cg20598190	Other	Intergeneic
6	32774788	cg22862357	Other	Intergeneic
5	36450470	ch.5.36486227R	Other	Intergeneic
12	73586034	ch.12.71872301F	Other	Intergeneic
12	49046495	ch.12.973812R	Other	Intergeneic
12	45356182	ch.12.902977F	Other	Intergeneic
10	44782092	cg10211193	Other	Intergeneic
11	86529655	cg11873113	Other	Intergeneic
14	22385791	cg27268120	Other	Intergeneic
3	116996975	cg10462597	Other	Intergeneic
14	78838275	ch.14.1202858F	Other	Intergeneic
6	127741813	cg12214090	Other	Intergeneic
16	49318747	cg26786800	Other	Intergeneic
5	53172915	ch.5.53208672R	Other	Intergeneic
6	139690828	cg18472160	Other	Intergeneic
11	22488445	cg17344099	Other	Intergeneic
7	142421812	cg19735804	Other	Intergeneic
5	1316636	cg10441424	Other	Intergeneic
11	121298154	cg13683424	Other	Intergeneic
X	16490374	ch.X.16400295F	Other	Intergeneic
8	130738823	cg17837330	Other	Intergeneic
13	54740236	cg15157312	Other	Intergeneic
6	29621467	cg14193550	Other	Intergeneic
4	43876927	cg21164813	Other	Intergeneic
5	12865512	cg16107470	Other	Intergeneic
10	120437770	ch.10.2535095F	Other	Intergeneic
16	65794720	cg10129884	Other	Intergeneic
8	40059435	cg20660197	Other	Intergeneic

2	70530862	cg17962671	Other	Intergeneic
5	6159585	cg09440150	Other	Intergeneic
10	132003854	cg13800652	Other	Intergeneic
8	49891730	cg08374859	Other	Intergeneic
14	35135441	cg23429457	Other	Intergeneic
13	58655819	cg09034331	Other	Intergeneic
5	97834710	ch.5.97862610R	Other	Intergeneic
7	23518135	cg10437900	Other	Intergeneic
1	18238511	ch.1.620704R	Other	Intergeneic
1	174947362	ch.1.173213985R	Other	Intergeneic
5	164258317	ch.5.3099968F	Other	Intergeneic
12	112849407	cg22190774	Other	Intergeneic
15	92065606	cg18774857	Other	Intergeneic
4	101220412	cg27216899	Other	Intergeneic
13	39068618	cg22937571	Other	Intergeneic

These CpGs were identified DNA methylation data from Yang (2014). The base position is based on National Centre for Biotechnology Information genome build 37.

Supplementary Table 2. List of CpG showing significant correlation with population doubling time of HCT116 cell lines

CpG	Location	Gene	PCC	FDR
cg14143055	Promoter	ADAMDEC1	0.91	0.01
cg21635584	Promoter	UBA3	0.9	0.01
cg24074685	Promoter	TIA1	0.88	0.02
cg07375367	Promoter	POLR1D	0.85	0.03
cg05782751	Promoter	MCTS1	0.81	0.05
ch.15.433532F	Body	RTF1	0.97	$1.6x10^{-3}$
ch.2.2207852R	Body	MRPS9	0.89	0.02
cg12803053	Body	NSMCE2	-0.87	0.02
ch.5.731560F	Body	ADAMTS12	0.86	0.03
ch.11.997072R	Body	MTCH2	0.86	0.03
ch.X.346519R	Body	CNKSR2	0.84	0.03
ch.1.1168472R	Body	THRAP3	0.84	0.04
ch.1.385573R	Body	PGD	0.84	0.04
ch.1.4018176R	Body	LGTN	0.83	0.04
cg22231602	Body	RGS7	0.83	0.04
ch.5.3268483F	Body	UBTD2	0.82	0.05
cg06422757	Other	PXT1	0.97	$1.73x10^{-3}$
cg10783042	Other	HDAC6	0.93	$6.20x10^{-3}$
cg25522119	Other	PRKD2	0.91	0.01
ch.22.436090R	Other	Intergeneic	0.9	0.02
ch.6.25083721F	Other	Intergeneic	0.88	0.02
ch.12.1700408R	Other	Intergeneic	0.88	0.02
ch.1.88799877F	Other	Intergeneic	0.87	0.02
cg05724777	Other	HLA-DQB1	0.87	0.02
ch.4.162336241R	Other	Intergeneic	0.87	0.02
cg27216899	Other	Intergeneic	-0.86	0.03
cg10413861	Other	Intergeneic	0.84	0.04
cg12210527	Other	ANAPC5	0.83	0.04
ch.21.284298R	Other	Intergeneic	0.83	0.04
cg13569146	Other	Intergeneic	0.83	0.04
cg19832312	Other	P4HA1	0.82	0.05
cg17550929	Other	Intergeneic	0.82	0.05
cg23429457	Other	Intergeneic	0.82	0.05
cg11159234	Other	Intergeneic	0.8	0.05

PCC: Pearson correlation coefficient. FDR: false discovery rate.

Supplementary Table 3. List of CpGs showing a strong correlation (absolute Pearson correlation coefficient ≥ 0.8) between DNA methylation and corresponding gene expression in HCT116 cell line

CpG	Gene	Location in the gene	PCC	P
cg17091793	FNDC3A	Promoter	1.00	7.30×10^{-4}
cg12019961	ATM	Promoter	-0.99	0.01
cg18959966	SMARCA1	Promoter	-0.98	0.02
cg07020846	TLE3	Promoter	0.98	0.02
cg23126949	ACCN2	Promoter	0.97	0.03
cg21388339	TP73	Promoter	-0.96	0.04
cg22865720	PRKCZ	Promoter	0.96	0.04
cg23548487	ZNF8	Promoter	0.95	0.05
cg17091793	FNDC3A	Promoter	-0.95	0.05
cg11404039	PLRG1	Promoter	-0.95	0.05
cg22603037	TCHH	Promoter	0.95	0.05
cg20664654	AVPR2	Promoter	0.94	0.06
cg15881990	PRPF18	Promoter	0.94	0.06
cg19113954	SEPSECS	Promoter	0.93	0.07
cg06484232	OR52E4	Promoter	-0.93	0.07
cg21472546	GABRB1	Promoter	0.93	0.07
cg19113954	SEPSECS	Promoter	0.93	0.07
cg11509733	TSPAN6	Promoter	-0.93	0.07
cg09817993	GNL3	Promoter	-0.93	0.07
cg10436877	ABCF2	Promoter	0.92	0.08
cg23193446	NMT2	Promoter	0.91	0.09
cg25198830	NGFRAP1	Promoter	-0.91	0.09
cg17060964	OR5AS1	Promoter	-0.91	0.09
cg10646076	COX7B	Promoter	0.90	0.10
cg26362852	RNF145	Promoter	-0.90	0.10
cg11785538	BCAP29	Promoter	-0.90	0.10
cg23903723	KIAA1949	Promoter	0.90	0.10
cg07375367	POLR1D	Promoter	-0.90	0.10
cg17012513	ARSF	Promoter	-0.89	0.11
cg15333689	SLU7	Promoter	0.89	0.11
cg16792062	ETS1	Promoter	0.89	0.11
cg27167381	MIR506	Promoter	0.89	0.11
cg26802063	ARSB	Promoter	-0.89	0.11
cg06148685	LPAR1	Promoter	0.89	0.11
cg18016370	PIGA	Promoter	-0.88	0.12
cg11509733	TSPAN6	Promoter	-0.87	0.13
cg12912949	ARVCF	Promoter	-0.87	0.13
cg06698332	S100A10	Promoter	0.87	0.13
cg13486082	NGFRAP1	Promoter	-0.87	0.13
cg26802063	ARSB	Promoter	0.87	0.13

cg18016370	PIGA	Promoter	-0.86	0.14
cg27379065	ZNF93	Promoter	-0.85	0.15
cg22865720	PRKCZ	Promoter	-0.85	0.15
cg18202167	OS9	Promoter	-0.85	0.15
cg26802063	ARSB	Promoter	0.85	0.15
cg25837979	MIR708	Promoter	-0.85	0.15
cg23620279	RPS24	Promoter	-0.83	0.17
cg10878114	SLC35F3	Promoter	0.82	0.18
cg25198830	NGFRAP1	Promoter	-0.82	0.18
cg18750937	CAND1	Promoter	0.82	0.18
cg14004049	TCEAL8	Promoter	0.82	0.18
cg23376554	TMSB4X	Promoter	-0.82	0.18
cg08097520	NTM	Promoter	0.82	0.18
cg17275074	TRPC7	Promoter	0.81	0.19
cg10561472	ZBTB7A	Promoter	-0.81	0.19
cg07151830	TCP1	Promoter	0.81	0.19
cg17952824	HNRNPA3P1	Promoter	-0.81	0.19
cg17952824	HNRNPA3P1	Promoter	-0.81	0.19
cg24787081	CDKN2AIP	Promoter	0.81	0.19
cg10828316	NRXN3	Promoter	-0.81	0.19
cg16253809	ARID5B	Promoter	0.80	0.20
cg23935054	OR4A5	Promoter	0.80	0.20
cg08099431	RORA	Body	1.00	1.75×10^{-5}
cg15794798	OBFC2A	Body	1.00	2.49×10^{-4}
ch.22.149158R	UBE2L3	Body	-1.00	6.18×10^{-4}
ch.17.79071R	SMYD4	Body	1.00	9.94×10^{-4}
cg11229715	CUL3	Body	-1.00	3.21×10^{-3}
ch.2.35699F	SNTG2	Body	-1.00	3.92×10^{-3}
cg06764736	SOX5	Body	1.00	4.33×10^{-3}
cg24806812	GC	Body	0.99	0.01
cg10069677	HELLS	Body	0.99	0.01
ch.8.362960F	GC	Body	0.99	0.01
cg13633856	MBNL3	Body	0.98	0.02
ch.1.2681285F	HIPK1	Body	0.98	0.02
cg13665890	SLC25A31	Body	0.98	0.02
cg11467638	SFRP2	Body	-0.97	0.03
cg23167425	ODZ2	Body	0.97	0.03
cg12949466	RGMB	Body	0.97	0.03
ch.3.343413R	NR2C2	Body	0.97	0.03
cg24132325	GC	Body	-0.96	0.04
ch.22.909671F	ATXN10	Body	-0.96	0.04
cg13689563	ZNF136	Body	0.96	0.04
ch.16.350833F	USP7	Body	0.96	0.04
ch.22.909671F	ATXN10	Body	-0.96	0.04
cg10116443	SLC22A9	Body	-0.95	0.05

ch.5.3268483F	UBTD2	Body	0.95	0.05
cg22834281	ZNF415	Body	0.95	0.05
cg21821990	SNX15	Body	-0.95	0.05
cg18495191	RPS6KA2	Body	-0.95	0.05
cg08247527	GC	Body	0.95	0.05
ch.3.343413R	NR2C2	Body	-0.95	0.05
cg15691035	LARS2	Body	0.94	0.06
cg25486361	KCNN2	Body	0.94	0.06
cg20124735	RORA	Body	0.94	0.06
cg16631432	SCN3A	Body	-0.93	0.07
ch.6.2623783F	MAP7	Body	-0.93	0.07
ch.4.1647744F	WDFY3	Body	-0.92	0.08
ch.4.1647744F	WDFY3	Body	-0.92	0.08
cg24132325	GC	Body	0.92	0.08
cg06103928	DLC1	Body	-0.92	0.08
ch.17.1348593F	MBTD1	Body	0.92	0.08
cg25497053	WWC3	Body	0.92	0.08
ch.12.1023240F	LIMA1	Body	0.92	0.08
cg23024358	SORCS1	Body	0.92	0.08
cg08247527	GC	Body	0.92	0.08
cg19753609	NRXN3	Body	-0.92	0.08
cg15651267	ODZ2	Body	0.92	0.08
cg15160274	GC	Body	0.92	0.08
cg15160274	SGCD	Body	0.92	0.08
ch.13.865492R	DIAPH3	Body	0.92	0.08
cg15160274	GC	Body	0.92	0.08
ch.11.1702122F	GAB2	Body	0.92	0.08
cg18495191	RPS6KA2	Body	-0.92	0.08
ch.1.2681285F	HIPK1	Body	-0.92	0.08
cg09236445	PDZRN3	Body	-0.91	0.09
ch.1.2681285F	HIPK1	Body	-0.91	0.09
cg23530596	SLC12A1	Body	0.90	0.10
ch.2.2207852R	MRPS9	Body	-0.90	0.10
ch.4.2245532F	PRDM5	Body	-0.90	0.10
cg07405178	RARB	Body	-0.90	0.10
ch.20.221631R	ANKRD5	Body	-0.89	0.11
cg12709692	KCTD8	Body	-0.89	0.11
cg23290313	ST3GAL3	Body	0.89	0.11
cg14520512	MBNL3	Body	0.89	0.11
cg09236445	PDZRN3	Body	-0.89	0.11
cg09811510	SCHIP1	Body	0.89	0.11
cg07491444	ARL6IP6	Body	-0.88	0.12
cg21478123	GMDS	Body	-0.88	0.12
ch.3.3822654R	ATP13A3	Body	-0.88	0.12
cg17165836	AFF3	Body	0.88	0.12

ch.1.953398R	TRNAU1AP	Body	-0.88	0.12
cg10626169	ABCA13	Body	0.88	0.12
cg18495191	RPS6KA2	Body	-0.88	0.12
ch.13.865492R	DIAPH3	Body	-0.88	0.12
cg13298997	PDE1C	Body	-0.88	0.12
cg18986048	MYPN	Body	-0.87	0.13
cg11019127	CADM1	Body	-0.87	0.13
cg25702790	GNAI1	Body	-0.87	0.13
ch.5.1443044F	AGGF1	Body	-0.87	0.13
cg15454195	NPAS3	Body	-0.87	0.13
ch.13.865492R	DIAPH3	Body	0.87	0.13
ch.9.1152820R	AGTPBP1	Body	-0.87	0.13
cg24132325	GC	Body	-0.86	0.14
ch.12.1019410F	LASS5	Body	0.84	0.16
ch.4.1647744F	WDFY3	Body	0.84	0.16
ch.4.1647744F	WDFY3	Body	0.84	0.16
ch.8.362960F	GC	Body	-0.84	0.16
ch.8.362960F	SGCZ	Body	-0.84	0.16
ch.4.2065340F	CCDC109B	Body	0.83	0.17
cg07491444	ARL6IP6	Body	-0.82	0.18
ch.20.1002962F	ZMYND8	Body	-0.82	0.18
cg07276831	FAT3	Body	0.82	0.18
cg24082347	ORAI1	Body	0.81	0.19
ch.8.903080R	WHSC1L1	Body	0.81	0.19
cg24806812	GC	Body	0.81	0.19
cg15459537	ARPP-21	Body	0.80	0.20
cg24132325	GC	Body	0.80	0.20
cg24132325	SGCD	Body	0.80	0.20
cg13361307	AFF3	Body	0.80	0.20
cg25609301	KCNH5	Body	0.80	0.20
ch.12.897509F	NELL2	Body	0.80	0.20
cg24492140	RFC3	Body	-0.80	0.20
ch.1.705736F	HP1BP3	Body	0.80	0.20
cg19210276	CTNND1	Other	-0.98	0.02
cg11113650	CCNB3	Other	0.98	0.02
ch.2.2007613R	CIAO1	Other	0.97	0.03
cg13208102	WBP5	Other	0.97	0.03
cg25385940	TTC23	Other	-0.97	0.03
cg19210276	CTNND1	Other	-0.96	0.04
cg17039262	SETD4	Other	0.96	0.04
ch.18.189560F	RAB12	Other	-0.96	0.04
cg22585269	CHEK2	Other	0.96	0.04
cg15558299	ELK4	Other	0.95	0.05
cg08155759	PEG3	Other	0.95	0.05
cg11920737	SERTAD2	Other	0.93	0.07

cg25385940	TTC23	Other	0.93	0.07
cg06422757	PXT1	Other	-0.93	0.07
cg07519822	GMPR2	Other	-0.93	0.07
cg21880712	ZNF136	Other	0.93	0.07
cg20522855	DMD	Other	-0.92	0.08
cg22585269	CHEK2	Other	-0.91	0.09
cg17039262	SETD4	Other	-0.91	0.09
cg27464574	WBP5	Other	0.91	0.09
cg19023258	ZNF493	Other	0.90	0.10
cg05723953	HMGB4	Other	-0.89	0.11
cg20522855	DMD	Other	0.88	0.12
cg24011341	SOX5	Other	0.88	0.12
ch.20.1062061F	STAU1	Other	0.87	0.13
cg08456555	ARHGAP6	Other	0.86	0.14
cg12931625	PI4K2B	Other	-0.85	0.15
cg09208571	MAGED2	Other	0.85	0.15
cg18132851	ESR1	Other	0.84	0.16
cg18198306	FBXO5	Other	-0.84	0.16
cg20528338	OSBPL1A	Other	-0.84	0.16
cg07099245	BCOR	Other	0.83	0.17
cg05724777	HLA-DQB1	Other	-0.83	0.17
cg19769080	PPM1B	Other	-0.81	0.19
cg26142661	ZMAT1	Other	-0.81	0.19
ch.10.820670F	KIF5B	Other	0.81	0.19
cg12962308	OR5D16	Other	-0.80	0.20
cg15830530	COX7B	Other	0.80	0.20
cg26856631	LSM5	Other	-0.80	0.20

PCC: Pearson correlation coefficient.

Supplementary Table 4. List of CpGs that showed strong correlation with gene expression and DNA methylation in HCT116 cell lines and showed differential methylated in TCGA colon cancer tissue as compare to adjacent healthy tissues

CpG	Location in the gene	Gene	Average methylation in cancer	Average methylation in normol	difference (Δβ)	FDR
cg10828316	Promoter	NRXN3	0.43	0.82	-0.39	$3.45x10^{-10}$
cg16792062	Promoter	ETS1	0.51	0.87	-0.36	$4.59x10^{-10}$
cg14597388	Promoter	GZMK	0.34	0.63	-0.29	$5.59x10^{-10}$
cg17060964	Promoter	OR5AS1	0.29	0.67	-0.38	$8.14x10^{-10}$
cg22603037	Promoter	TCHH	0.55	0.87	-0.32	$1.60x10^{-9}$
cg22155405	Promoter	LOC284441	0.45	0.75	-0.30	$4.05x10^{-9}$
cg05788138	Promoter	OR5L1	0.30	0.56	-0.26	$4.85x10^{-9}$
cg14640659	Promoter	PCDHB13	0.32	0.58	-0.27	$6.30x10^{-9}$
cg10878114	Promoter	SLC35F3	0.68	0.43	0.25	$6.51x10^{-8}$
cg23935054	Promoter	OR4A5	0.13	0.21	-0.07	$1.81x10^{-7}$
cg24109012	Promoter	ZNF709	0.53	0.79	-0.26	$2.06x10^{-7}$
cg14696870	Promoter	FCER1A	0.57	0.86	-0.29	$7.63x10^{-7}$
cg23434090	Promoter	OR51L1	0.74	0.90	-0.16	$2.33x10^{-6}$
cg26155802	Promoter	OR4F6	0.64	0.87	-0.24	$5.84x10^{-6}$
cg08060810	Promoter	OR5D18	0.77	0.92	-0.15	$3.74x10^{-5}$
cg16395953	Promoter	IFIT1	0.09	0.07	0.02	$1.24x10^{-4}$
cg09073052	Promoter	PLEKHM2	0.15	0.11	0.03	2.85E-04
cg17952824	Promoter	HNRNPA3P1	0.38	0.52	-0.14	$6.25x10^{-4}$
cg14276619	Promoter	HRK	0.28	0.17	0.10	$6.42x10^{-4}$
cg13486082	Promoter	NGFRAP1	0.78	0.90	-0.13	$1.04x10^{-3}$
cg27379065	Promoter	ZNF93	0.15	0.09	0.06	$1.16x10^{-3}$
cg13053563	Promoter	OR2T1	0.73	0.86	-0.12	$1.58x10^{-3}$
cg05884522	Promoter	NOL7	0.05	0.06	-0.01	$2.21x10^{-3}$
cg26362852	Promoter	RNF145	0.15	0.11	0.04	$2.50x10^{-3}$
cg22845496	Promoter	UNC13C	0.74	0.87	-0.13	$2.74x10^{-3}$
cg20485144	Promoter	ID3	0.15	0.12	0.03	$3.78x10^{-3}$
cg07020846	Promoter	TLE3	0.09	0.08	0.01	$3.84x10^{-3}$
cg23548487	Promoter	ZNF8	0.08	0.04	0.04	$4.62x10^{-3}$
cg07312099	Promoter	ASAH1	0.13	0.10	0.03	$5.46x10^{-3}$
cg13675958	Promoter	C13orf27	0.09	0.04	0.05	$7.44x10^{-3}$
cg11471799	Promoter	DIXDC1	0.14	0.10	0.04	$7.74x10^{-3}$
cg13108328	Promoter	PLEKHA5	0.06	0.07	-0.01	$8.85x10^{-3}$
cg11785538	Promoter	BCAP29	0.24	0.29	-0.05	0.01
cg25016070	Promoter	PKM2	0.13	0.10	0.02	0.01
cg25198830	Promoter	NGFRAP1	0.77	0.87	-0.10	0.01
cg17012513	Promoter	ARSF	0.21	0.24	-0.03	0.02
cg12583184	Promoter	SLC24A6	0.23	0.19	0.03	0.03

cg17275074	Promoter	TRPC7	0.23	0.24	-0.01	0.03
cg07494499	Body	NXN	0.36	0.69	-0.34	$3.45x10^{-10}$
cg24132325	Body	SGCD	0.44	0.85	-0.41	$3.45x10^{-10}$
cg23530596	Body	SLC12A1	0.27	0.62	-0.35	$3.92x10^{-10}$
cg15651267	Body	ODZ2	0.43	0.83	-0.40	$4.05x10^{-10}$
cg16522250	Body	CNTN6	0.39	0.84	-0.44	$6.83x10^{-10}$
cg21222888	Body	GPC6	0.23	0.52	-0.29	$6.83x10^{-10}$
cg19017553	Body	PARD3	0.46	0.79	-0.33	$6.83x10^{-10}$
cg06707406	Body	NRXN1	0.44	0.79	-0.36	$8.14x10^{-10}$
cg17937340	Body	COL6A6	0.56	0.85	-0.29	$8.28x10^{-10}$
cg07977614	Body	PLD5	0.17	0.51	-0.33	$8.76x10^{-10}$
cg22092126	Body	AFF3	0.39	0.75	-0.35	$9.82x10^{-10}$
cg24806812	Body	GC	0.60	0.88	-0.28	$4.85x10^{-9}$
cg16253976	Body	ADCY2	0.45	0.80	-0.35	$5.30x10^{-9}$
cg19567866	Body	SPOCK1	0.50	0.85	-0.36	$5.30x10^{-9}$
cg16051561	Body	CTNND2	0.30	0.61	-0.30	$6.30x10^{-9}$
cg18413062	Body	OPCML	0.36	0.67	-0.30	$8.82x10^{-9}$
cg23024358	Body	SORCS1	0.65	0.83	-0.18	$9.70x10^{-9}$
cg17519477	Body	NRG3	0.25	0.52	-0.26	$9.83x10^{-9}$
cg09811510	Body	SCHIP1	0.31	0.67	-0.36	$1.01x10^{-8}$
cg16631432	Body	SCN3A	0.46	0.77	-0.31	$1.84x10^{-8}$
cg25361651	Body	DEFB115	0.15	0.29	-0.13	$3.40x10^{-8}$
cg10626169	Body	ABCA13	0.48	0.81	-0.34	$4.82x10^{-8}$
cg22834281	Body	ZNF415	0.64	0.92	-0.28	$5.98x10^{-8}$
cg23290313	Body	ST3GAL3	0.53	0.79	-0.26	$6.40x10^{-8}$
cg27583655	Body	PDE4D	0.40	0.63	-0.24	$6.97x10^{-8}$
cg19753609	Body	NRXN3	0.52	0.79	-0.28	$8.65x10^{-8}$
cg07467482	Body	DPP6	0.49	0.79	-0.30	$1.81x10^{-7}$
cg08238568	Body	RGS7	0.58	0.83	-0.25	$2.16x10^{-7}$
cg26349484	Body	SORCS3	0.24	0.43	-0.18	$7.27x10^{-7}$
cg19093405	Body	CACNA2D3	0.78	0.91	-0.13	$1.56x10^{-7}$
cg08099431	Body	RORA	0.73	0.91	-0.18	$3.11x10^{-6}$
cg18818949	Body	NRXN3	0.38	0.60	-0.22	$3.17x10^{-6}$
cg15459537	Body	ARPP-21	0.35	0.53	-0.17	$1.31x10^{-5}$
cg15826891	Body	LOC399959	0.57	0.76	-0.19	$1.83x10^{-5}$
cg12033248	Body	KCNU1	0.65	0.88	-0.23	$2.67x10^{-5}$
cg11458498	Body	SLIT3	0.39	0.55	-0.16	$6.15x10^{-5}$
cg06764736	Body	SOX5	0.84	0.93	-0.10	$8.88x10^{-5}$
cg25461513	Body	CADM1	0.82	0.90	-0.08	$1.33x10^{-4}$
cg16819888	Body	AUTS2	0.78	0.91	-0.13	$1.49x10^{-4}$
cg10116443	Body	SLC22A9	0.73	0.91	-0.18	$1.62x10^{-4}$
cg15794798	Body	OBFC2A	0.14	0.11	0.04	$1.67x10^{-4}$
cg22359828	Body	CACNA1E	0.22	0.31	-0.09	$2.85x10^{-4}$
cg25486361	Body	KCNN2	0.69	0.82	-0.13	$3.06x10^{-4}$
cg06103928	Body	DLC1	0.74	0.86	-0.11	$3.77x10^{-4}$

cg16242106	Body	UBE2Q2	0.17	0.12	0.06	$3.77x10^{-4}$
cg09566894	Body	TSHZ2	0.67	0.87	-0.20	$4.07x10^{-4}$
cg16752940	Body	LSAMP	0.81	0.90	-0.09	$5.37x10^{-4}$
ch.20.1002962F	Body	ZMYND8	0.17	0.23	-0.06	$7.60x10^{-4}$
cg22872195	Body	UNC5D	0.41	0.56	-0.15	$1.30x10^{-3}$
cg14773588	Body	PHACTR1	0.75	0.86	-0.11	$1.34x10^{-3}$
cg07405178	Body	RARB	0.19	0.14	0.05	$1.67x10^{-3}$
cg19053479	Body	FAM188A	0.16	0.13	0.03	$2.45x10^{-3}$
ch.22.909671F	Body	ATXN10	0.14	0.10	0.04	$2.50x10^{-3}$
ch.4.1647744F	Body	WDFY3	0.10	0.07	0.03	$2.62x10^{-3}$
ch.3.343413R	Body	NR2C2	0.10	0.07	0.03	$3.10x10^{-3}$
ch.4.2245532F	Body	PRDM5	0.09	0.07	0.02	$4.25x10^{-3}$
cg12708807	Body	CRYZL1	0.10	0.08	0.02	$4.34x10^{-3}$
ch.8.343778F	Body	DLC1	0.14	0.10	0.04	$6.29x10^{-3}$
cg16758887	Body	LOC93622	0.12	0.09	0.02	$8.01x10^{-3}$
cg22892110	Body	MAPK15	0.26	0.16	0.10	$8.43x10^{-3}$
cg21324884	Body	MIR548A2	0.62	0.76	-0.14	$9.40x10^{-3}$
cg14520512	Body	MBNL3	0.59	0.70	-0.11	0.01
cg06938601	Body	TCERG1L	0.37	0.45	-0.08	0.01
cg10069677	Body	HELLS	0.14	0.11	0.03	0.02
cg06721860	Body	SYT10	0.79	0.87	-0.08	0.02
cg21518865	Body	ST8SIA3	0.47	0.57	-0.10	0.02
ch.1.1168472R	Body	THRAP3	0.08	0.07	0.01	0.02
ch.1.705736F	Body	HP1BP3	0.12	0.09	0.03	0.02
cg07097925	Body	ICAM5	0.10	0.04	0.06	0.03
cg25486749	Body	TCERG1L	0.89	0.94	-0.04	0.04
ch.2.4215183F	Body	CPS1	0.10	0.08	0.02	0.04
ch.15.433532F	Body	RTF1	0.19	0.17	0.02	0.04
cg07293993	Body	MTHFS	0.17	0.14	0.03	0.04

FDR: False discovery rate. Wilcoxon non-parametric test was used to calculate the p value.

Supplementary Table 5. Differential expression analysis of genes corresponding to differentially methylated CpGs at promoter and gene body area in TCGA colon cancer data

Gene	CpG	Location in the gene	Average expression in cancer	Average expression in normol	Fold change	FDR
FCER1A	cg14696870	Promoter	-0.20	1.33	-1.52	8.63×10^{-9}
NOL7	cg05884522	Promoter	0.18	-1.21	1.38	1.13×10^{-8}
C13orf27	cg13675958	Promoter	0.15	-1.09	1.25	2.43×10^{-8}
PKM2	cg25016070	Promoter	0.19	-1.16	1.35	2.43×10^{-8}
HNRNPA3P1	cg17952824	Promoter	0.16	-1.19	1.36	2.33×10^{-7}
ID3	cg20485144	Promoter	-0.12	1.01	-1.12	4.14×10^{-6}
ZNF8	cg23548487	Promoter	0.15	-0.84	1.00	7.29×10^{-6}
SLC24A6	cg12583184	Promoter	-0.12	0.80	-0.92	1.43×10^{-5}
SLC35F3	cg10878114	Promoter	-0.10	0.81	-0.92	2.37×10^{-5}
DIXDC1	cg11471799	Promoter	-0.14	0.92	-1.07	2.53×10^{-5}
IFIT1	cg16395953	Promoter	-0.11	0.67	-0.77	1.08×10^{-3}
PLEKHA5	cg13108328	Promoter	0.07	-0.56	0.63	1.29×10^{-3}
TLE3	cg07020846	Promoter	-0.06	0.49	-0.55	4.95×10^{-3}
ZNF93	cg27379065	Promoter	0.06	-0.30	0.36	4.95×10^{-3}
ASAH1	cg07312099	Promoter	-0.06	0.57	-0.63	0.01
ETS1	cg16792062	Promoter	-0.06	0.54	-0.60	0.01
ZNF709	cg24109012	Promoter	0.04	-0.41	0.45	0.01
BCAP29	cg11785538	Promoter	-0.06	0.42	-0.48	0.03
GZMK	cg14597388	Promoter	-0.06	0.45	-0.51	0.03
HELLS	cg10069677	Genebody	0.26	-1.64	1.90	3.98×10^{-10}
NRXN1	cg06707406	Genebody	-0.24	1.70	-1.94	3.98×10^{-10}
UNC5D	cg22872195	Genebody	-0.23	1.59	-1.82	3.98×10^{-10}
CTNND2	cg16051561	Genebody	-0.25	1.69	-1.94	5.10×10^{-10}
SCN3A	cg16631432	Genebody	-0.17	1.27	-1.44	5.53×10^{-10}
MAPK15	cg22892110	Genebody	0.20	-1.35	1.55	8.40×10^{-10}
SORCS1	cg23024358	Genebody	-0.20	1.45	-1.66	1.08×10^{-9}
DPP6	cg07467482	Genebody	-0.24	1.48	-1.72	1.25×10^{-9}
AFF3	cg22092126	Genebody	-0.21	1.28	-1.48	2.75×10^{-9}
PDE4D	cg27583655	Genebody	-0.15	1.20	-1.35	1.31×10^{-8}
SLIT3	cg11458498	Genebody	-0.17	1.27	-1.44	1.31×10^{-8}
ZNF415	cg22834281	Genebody	-0.15	1.08	-1.22	1.15×10^{-6}
ADCY2	cg16253976	Genebody	-0.14	1.02	-1.17	2.07×10^{-6}
ST3GAL3	cg23290313	Genebody	-0.15	0.99	-1.13	2.35×10^{-6}
NRG3	cg17519477	Genebody	-0.17	1.12	-1.29	2.54×10^{-6}
CRYZL1	cg12708807	Genebody	-0.17	0.91	-1.07	4.11×10^{-6}
ZMYND8	ch.20.1002962F	Genebody	0.13	-0.94	1.07	4.85×10^{-6}
MBNL3	cg13633856	Genebody	-0.12	0.81	-0.93	1.73×10^{-5}
CACNA2D3	cg19093405	Genebody	-0.12	0.87	-0.98	3.21×10^{-5}
LOC93622	cg16758887	Genebody	0.13	-0.69	0.82	5.30×10^{-5}

SOX5	cg06764736	Genebody	-0.11	0.80	-0.91	$8.41x10^{-5}$
ODZ2	cg23167425	Genebody	-0.10	0.72	-0.82	$1.76x10^{-4}$
CADM1	cg11019127	Genebody	-0.08	0.67	-0.75	$5.54x10^{-4}$
AUTS2	cg09703727	Genebody	0.07	-0.40	0.46	$5.76x10^{-4}$
GPC6	cg21222888	Genebody	-0.09	0.75	-0.85	$5.76x10^{-4}$
LOC399959	cg15826891	Genebody	-0.10	0.76	-0.85	$6.31x10^{-4}$
RORA	cg08099431	Genebody	-0.07	0.63	-0.71	$1.91x10^{-3}$
THRAP3	ch.1.1168472R	Genebody	0.10	-0.65	0.76	$2.24x10^{-3}$
ATXN10	ch.22.909671F	Genebody	0.08	-0.57	0.65	$2.37x10^{-3}$
SCHIP1	cg09811510	Genebody	-0.07	0.72	-0.80	$4.40x10^{-3}$
TCERG1L	cg06938601	Genebody	-0.07	0.64	-0.71	0.01
DLC1	cg06103928	Genebody	-0.06	0.65	-0.71	0.01
OBFC2A	cg15794798	Genebody	0.10	-0.51	0.61	0.01
PHACTR1	cg14773588	Genebody	-0.05	0.48	-0.53	0.02
MTHFS	cg07293993	Genebody	0.07	-0.39	0.46	0.04
ICAM5	cg07097925	Genebody	0.10	-0.44	0.54	0.04
COL6A6	cg17937340	Genebody	0.08	-0.43	0.51	0.05
SGCD	cg24132325	Genebody	-0.05	0.50	-0.54	0.05
FCER1A	cg14696870	Promoter	-0.20	1.33	-1.52	$8.63x10^{-9}$
NOL7	cg05884522	Promoter	0.18	-1.21	1.38	$1.13x10^{-8}$
C13orf27	cg13675958	Promoter	0.15	-1.09	1.25	$2.43x10^{-8}$
PKM2	cg25016070	Promoter	0.19	-1.16	1.35	$2.43x10^{-8}$
HNRNPA3P1	cg17952824	Promoter	0.16	-1.19	1.36	$2.33x10^{-7}$
ID3	cg20485144	Promoter	-0.12	1.01	-1.12	$4.14x10^{-6}$
ZNF8	cg23548487	Promoter	0.15	-0.84	1.00	$7.29x10^{-6}$
SLC24A6	cg12583184	Promoter	-0.12	0.80	-0.92	$1.43x10^{-5}$
SLC35F3	cg10878114	Promoter	-0.10	0.81	-0.92	$2.37x10^{-5}$
DIXDC1	cg11471799	Promoter	-0.14	0.92	-1.07	$2.53x10^{-5}$
IFIT1	cg16395953	Promoter	-0.11	0.67	-0.77	$1.08x10^{-3}$
PLEKHA5	cg13108328	Promoter	0.07	-0.56	0.63	$1.29x10^{-3}$
TLE3	cg07020846	Promoter	-0.06	0.49	-0.55	$4.95x10^{-3}$
ZNF93	cg27379065	Promoter	0.06	-0.30	0.36	$4.95x10^{-3}$
ASAH1	cg07312099	Promoter	-0.06	0.57	-0.63	0.01
ETS1	cg16792062	Promoter	-0.06	0.54	-0.60	0.01
ZNF709	cg24109012	Promoter	0.04	-0.41	0.45	0.01
BCAP29	cg11785538	Promoter	-0.06	0.42	-0.48	0.03
GZMK	cg14597388	Promoter	-0.06	0.45	-0.51	0.03

FDR: False discovery rate. Wilcoxon non-parametric test was used to calculate the p value.

Supplementary Figure 1. Box plot showing a decrease in methylation level of loci other than identified CpGs in T24 and MCF7 cell line after decitabine treatment.

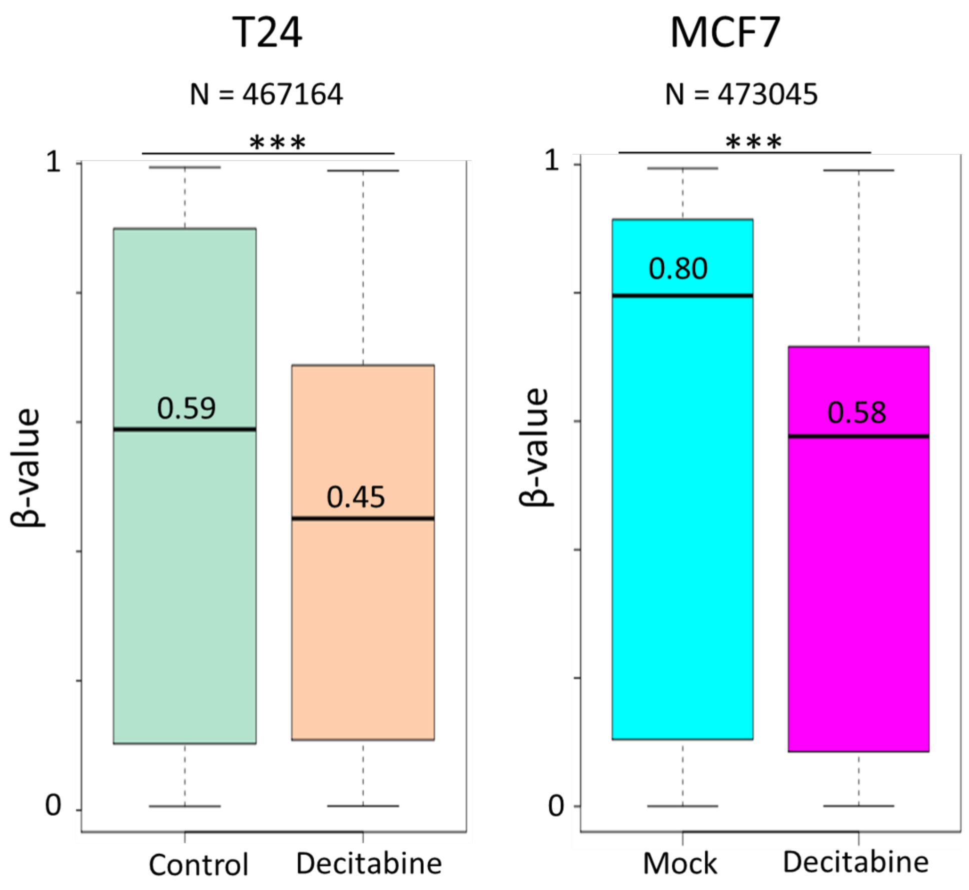

N denotes the total number of CpGs analyzed in the data. The data from the study by Han et al (GSE41525) and Leadem et al (GSE97483) are shown for T24 and MCF7 cells, respectively.

Supplementary Figure 2. Comparison of methylation levels of identified CpGs in decitabine treated and untreated 14 PBMCs (P1-P14), 8 tumor biopsies (T1-T8) and 6 ascites (A1-A6) samples of platinum resistant epithelial ovarian cancer patients.

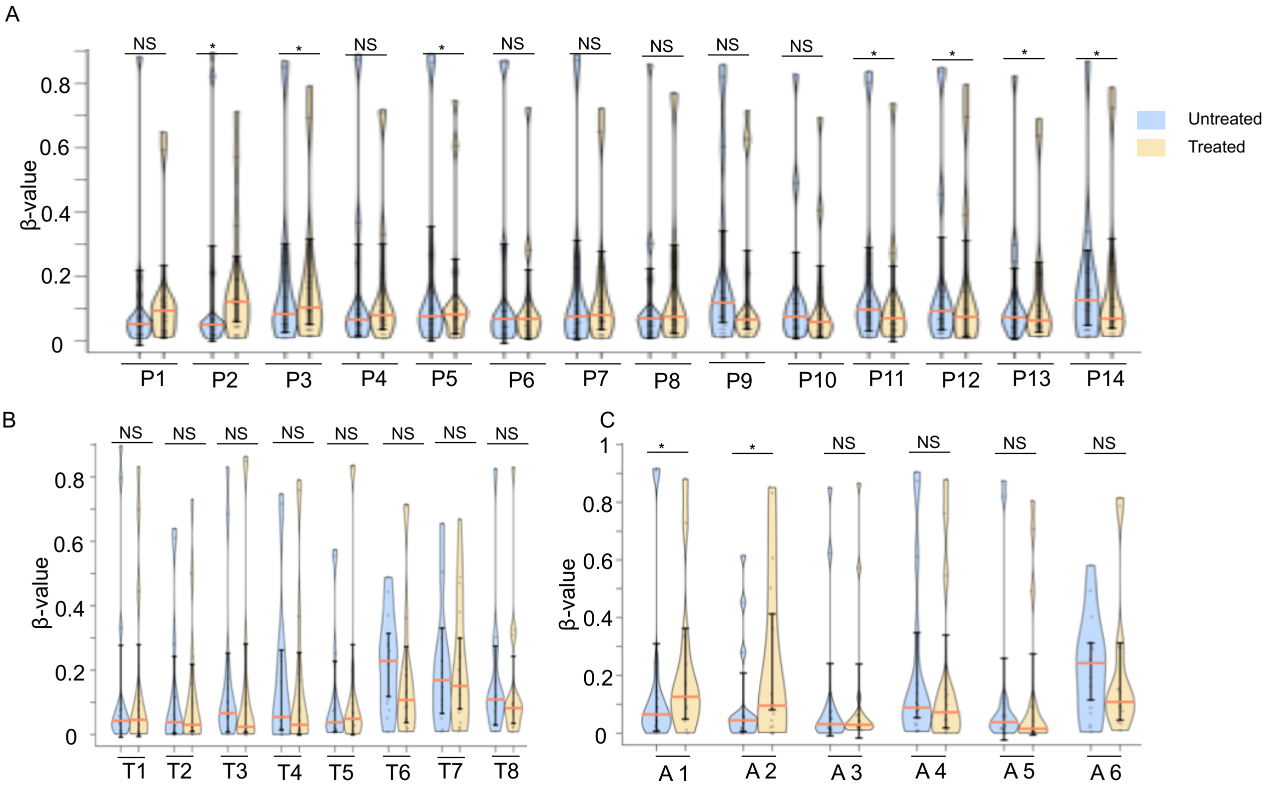

P denotes peripheral blood mononuclear cells, T tumor biopsy samples, and A ascites.

Pirate plot comparing the methylation profile of untreated (day 1) and decitabine treated (day 8) samples. PBMCs (A), tumor biopsy (B), and ascites (C) samples from recurrent platinum resistance ovarian cancer patients. Patients have been treated with decitabine (Eisai) at 10 mg/m^2 given intravenously daily for 5 days. The comparison has been made on the basis of 16 common CpGs out of 638 hypermethylated CpGs present in the data. The pirate plot shows the median methylation level (horizontal color line) and distribution pattern (mean ± standard error as vertical black line) of the 16 CpGs. The statistical significance was assessed using the non-parametric Wilcoxon signed-rank test. *$p < 0.05$, NS= Not significant

Supplementary Figure 3. Enrichment analysis of identified CpGs in enhancer and regulatory regions.

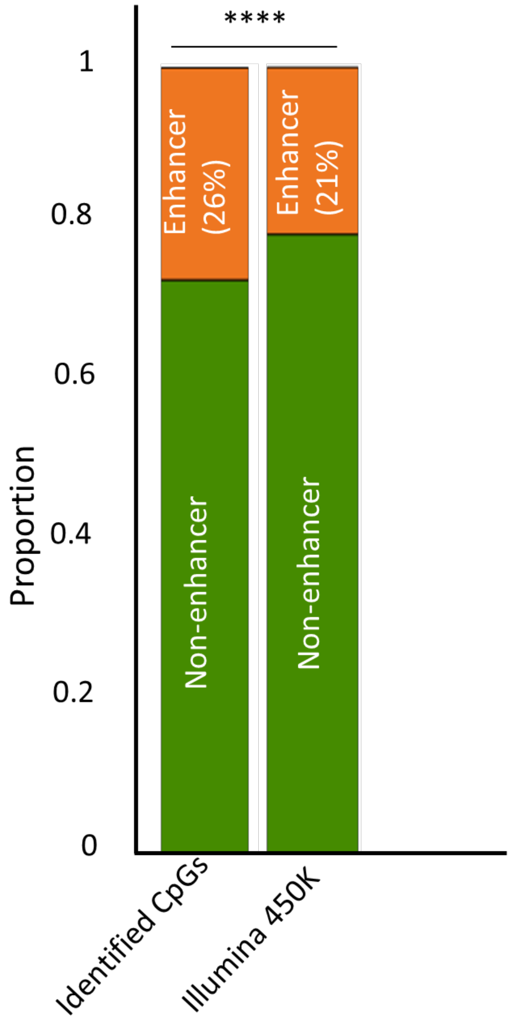

Eenrichment of identified CpGs in enhancer region of the genome. CpGs with increased methylations were enriched in enhancer region (21% of identified CpGs were in enhancer region as compared to 26% of total CpGs present in 450K chip, P = 3.36×10^{-4}). The proportion of identified CpGs in enhancer region is shown as segmented barplot (upper segment). ***P<0.0005.

Supplementary Figure 4. Interaction network of genes regulated by NFATc1.

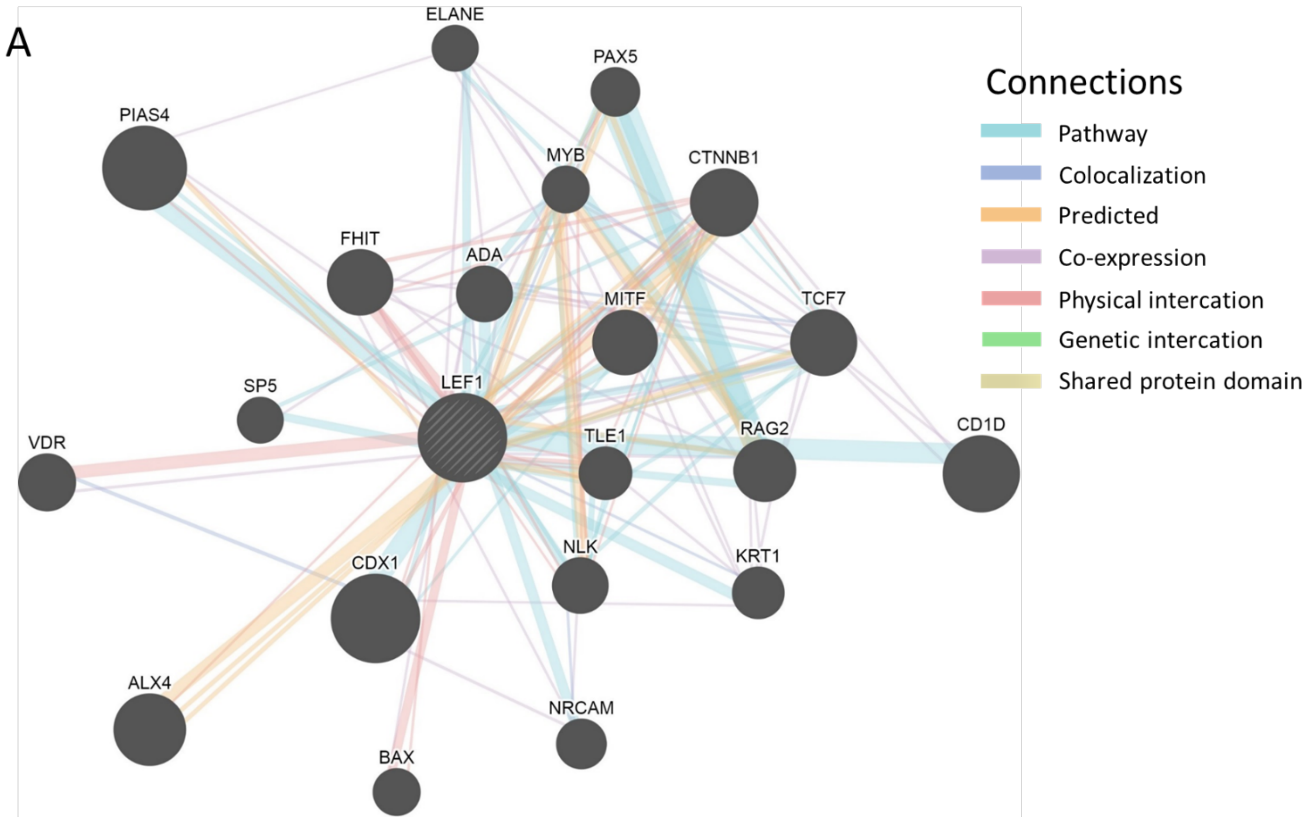

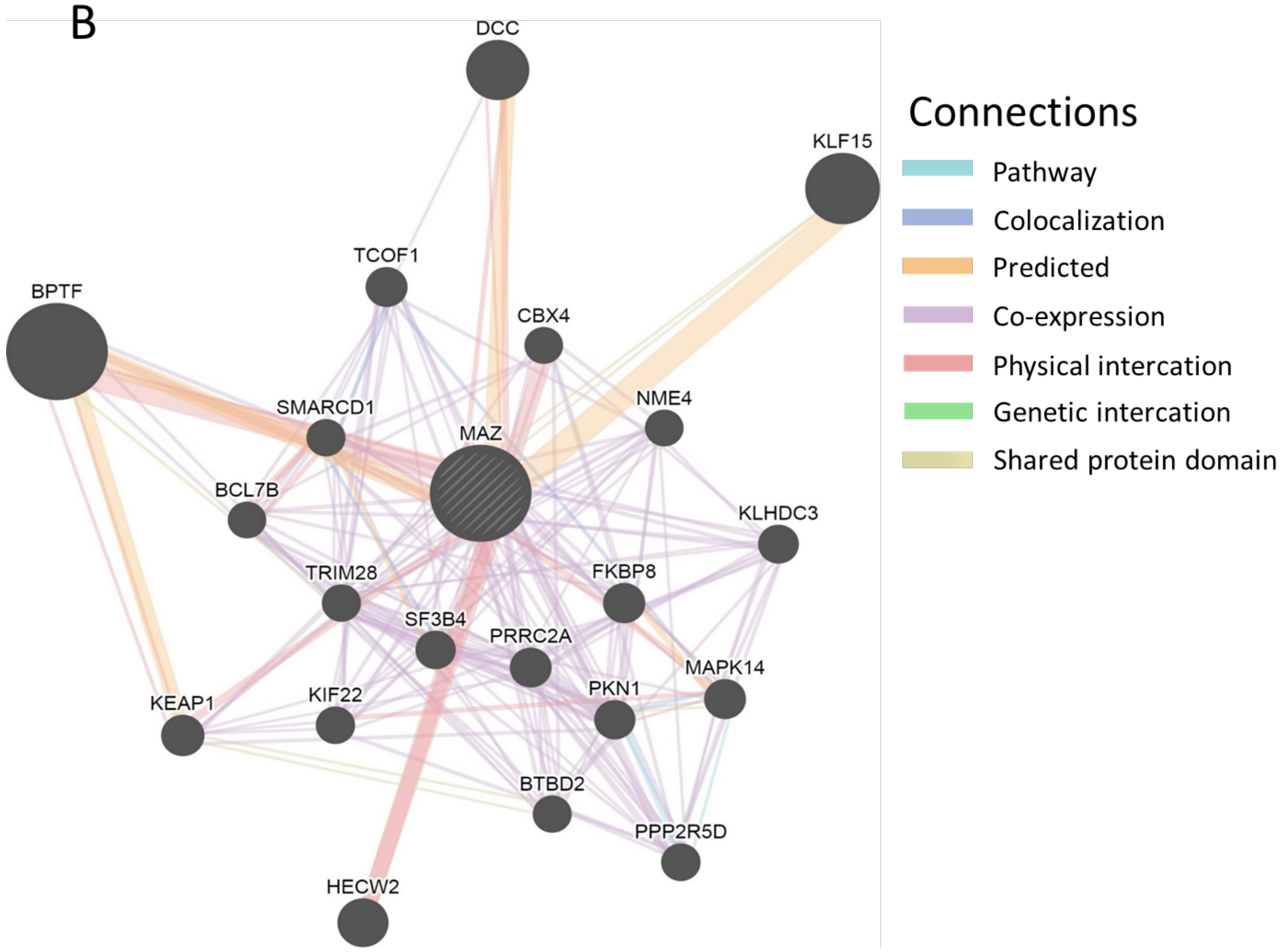
B
DCC
KLF15
TCOF1
BPTF
CBX4
NME4
SMARCD1
MAZ
BCL7B
KLHDC3
TRIM28
FKBP8
SF3B4
PRRC2A
MAPK14
KEAP1
KIF22
PKN1
BTBD2
PPP2R5D
HECW2
Connections
Pathway
Colocalization
Predicted
Co-expression
Physical intercation
Genetic intercation
Shared protein domain

C

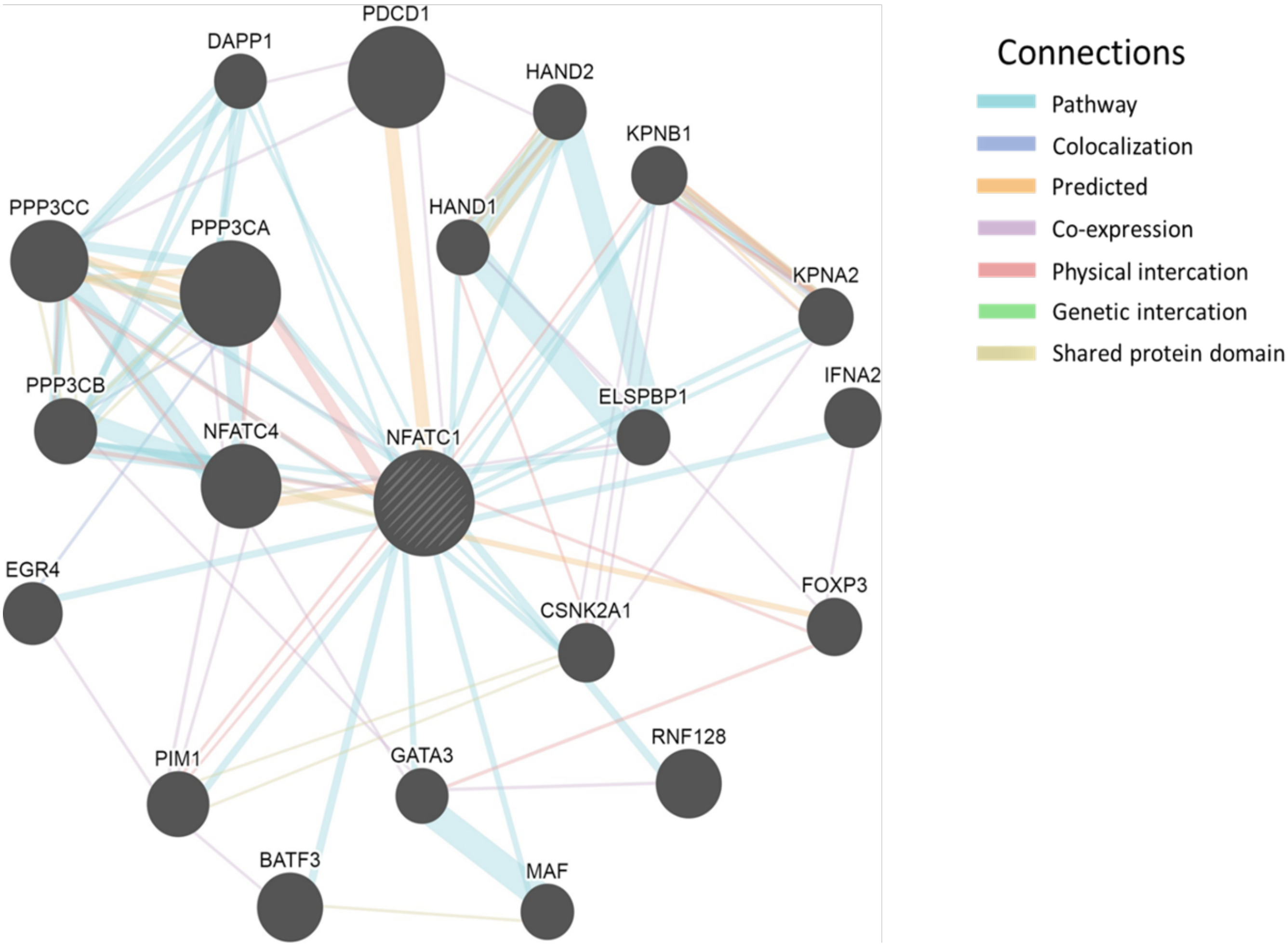

Only interactions among those genes that are directly connected to LEF1 (A), MAZ (B), and NFATc1 (C) have been shown using network construction from GENEMANIA. The size of the nodes is proportional to gene score calculated by GENEMANIA using label propagation algorithm that indicates the relevance of each gene to the original list based on the selected networks.

Supplementary Figure 5. Comparison of the expression levels of DNA methylating enzymes (DNMT3A, DNMT3B, DNMT1 and DNMTL) in 26 breast, 12 colorectal cancer and 13 ovarian cancer cell (Li H. et al., 2014) lines after 72 hours of low dose (0.5 μM) azacytidine treatment.

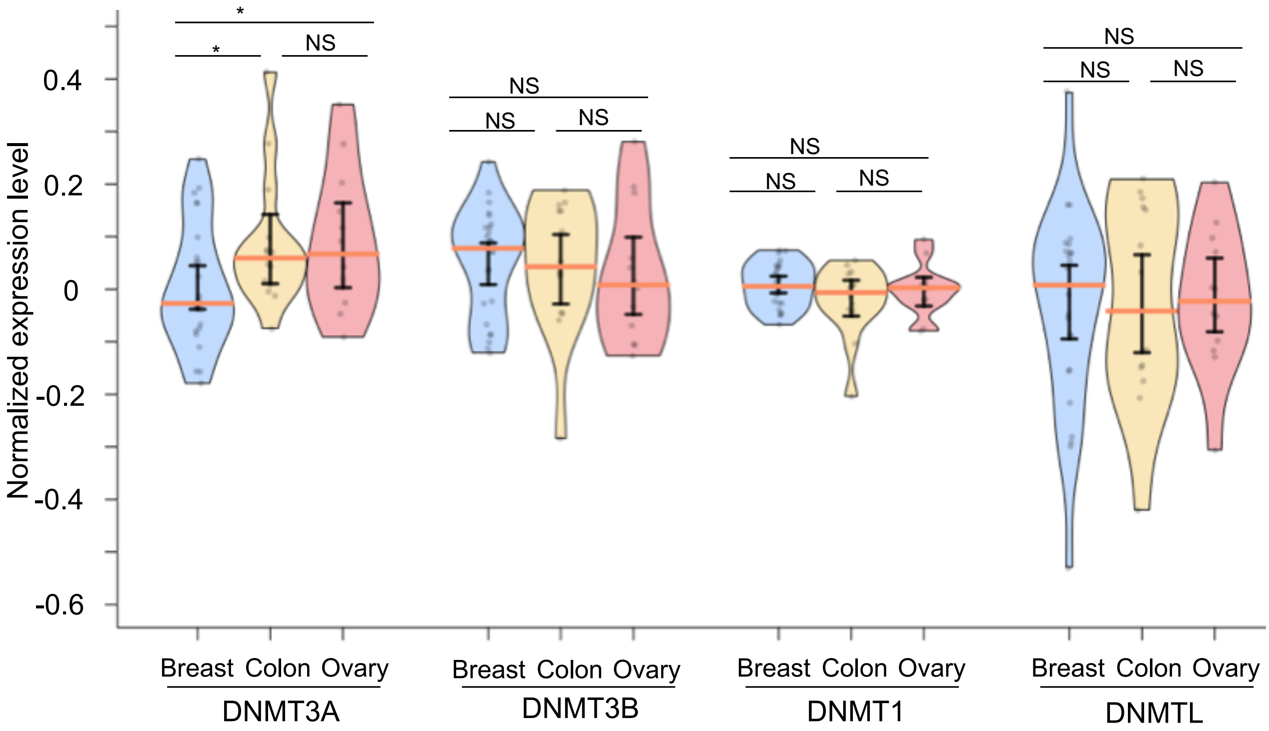

Pirate plots show the median methylation levels (horizontal colored line) and distribution pattern (mean ± standard error as vertical black line) of the DNA methylating enzymes (DNMT3A, DNMT3B, DNMT1 and DNMT3L). NS= Not significant *p<0.05. P value has been calculated using nonparametric Wilcoxon test.
